# CAMKK2 restored mitochondrial dynamics homeostasis to alleviate pulmonary fibrosis via AMPK/PGC-1α signaling pathway in lung fibroblasts

**DOI:** 10.1186/s10020-025-01373-5

**Published:** 2025-10-06

**Authors:** Yanlin Zhou, Yuqi Wang, Mengyuan Wang, Bin Li, Kai Xu, Xiaoyue Pan, Zhongzheng Li, Qiwen Wang, Wanyu He, Jiaojiao Pang, Yingchun Guo, Yuqing Zhang, Koji Sakamoto, Juntang Yang, Lan Wang, Guoying Yu

**Affiliations:** 1 State Key Laboratory of Cell Differentiation and Regulation, Henan International Joint Laboratory of Pulmonary Fibrosis, Henan Center for Outstanding Overseas Scientists of Organ Fibrosis, Pingyuan Laboratory, College of Life Science, Henan Normal university, Xinxiang, 453007 China; 2 College of Basic Medicine, North Henan Medical University, Xinxiang, 453003 China; 3 Department of Respiratory Medicine, Nagoya University Graduate School of Medicine, Nagoya, 466–8550 Japan

**Keywords:** CAMKK2, Mitochondrial dynamics, Energy metabolism, Fibroblasts, Pulmonary fibrosis

## Abstract

**Background:**

Idiopathic pulmonary fibrosis (IPF) is a fatal lung disease characterized by aberrant fibroblast activation and extracellular matrix deposition. Emerging evidence implicates mitochondrial dysfunction in IPF pathogenesis. Although CAMKK2 has been implicated in mitochondrial function associated with diabetic nephropathy, its role and underlying mechanisms in IPF remain unclear. This study aims to investigate the role of CAMKK2 in IPF.

**Methods:**

This study employed AAV-CAMKK2 mice, primary human/mouse lung fibroblasts, MRC-5 cells, and IPF patient samples. The CAMKK2 inhibitor (STO-609), shCAMKK2 and Flag-CAMKK2 overexpression plasmid were used to investigate the role of CAMKK2 in lung fibroblasts. Fibroblast activity was assessed by transwell migration, collagen contraction, and wound-healing assays. Mitochondrial function (ROS, mitochondrial membrane potential (MMP), ATP and oxygen consumption rate (OCR)) was measured by ROS assay, JC-1 assay, ATP assay and Seahorse assay. Mitochondrial dynamics (MFN1/MFN2, DRP1), PGC-1α, and p-^T172^AMPK were analyzed alongside fibrotic markers (FN1, COL1A1 and α-SMA) by Western blotting and RT-qPCR. The AMPK inhibitor (Compound C) and PGC-1α inhibitor (SR-18292) were used to investigate the role of AMPK and PGC-1α in this study.

**Results:**

This study revealed that CAMKK2 expression was significantly downregulated in pulmonary fibrosis, concomitant with impaired mitochondrial function-related proteins (PGC-1α, MFN1and MFN2). In vitro experiments demonstrated that CAMKK2 inhibition or shCAMKK2 knockdown exacerbated fibroblast activation and extracellular matrix (ECM) production in both MRC-5 cells and primary mouse lung fibroblasts. Conversely, CAMKK2 overexpression attenuated TGF-β1-induced fibroblast activation and ECM deposition in MRC-5 cells and primary human lung fibroblasts. Further investigation established that CAMKK2 overexpression normalized mitochondrial morphology, enhanced MMP, ATP content and respiratory capacity to ameliorate mitochondrial dysfunction in TGF-β1–induced MRC-5 cells. Moreover, CAMKK2 upregulated mitochondrial fusion protein expression (MFN1 and MFN2) and suppressed fission (DRP1) in MRC-5 cells. Mechanistically, CAMKK2 regulated mitochondrial dynamics and OXPHOS function via the AMPK/PGC-1α pathway in TGF-β1–induced MRC-5 cells, as evidenced by rescued protein expression (MFN1 and MFN2), and reversal of these effects following AMPK or PGC-1α inhibition. In vivo studies showed that AAV-mediated CAMKK2 delivery significantly attenuated bleomycin-induced pulmonary fibrosis in mice.

**Conclusions:**

In summary, these findings collectively demonstrate that CAMKK2 regulated mitochondrial dynamics and OXPHOS function via AMPK/PGC-1α signaling pathway to alleviate pulmonary fibrosis in lung fibroblasts. Therefore, targeting CAMKK2 presents a novel and promising therapeutic strategy for the treatment of pulmonary fibrosis.

**Supplementary Information:**

The online version contains supplementary material available at 10.1186/s10020-025-01373-5.

## Introduction

IPF is a chronic, progressive lung disease characterized by scarring of lung tissue, severely impacting both quality of life and prognosis(Podolanczuk et al. 2023; Bonella et al. 2023). A hallmark of pulmonary fibrosis is the excessive activation of fibroblasts and over-deposition of extracellular matrix (ECM), leading to the destruction of lung architecture and loss of function(Spagnolo et al. 2021; Adegunsoye et al. 2024). Scar formation is driven by factors such as TGF-β1, which stimulates the active metabolism of fibroblasts, influenced by biomechanical and biochemical conditions(Rangarajan et al. 2018). Typically, the median survival duration for IPF is between 3 and 5 years. Although treatments like pirfenidone and nintedanib can slow down disease progression without cure. However, the underlying mechanisms of pulmonary fibrosis remain unclear, and thus new therapeutic approaches are imperative.

Dysfunctional mitochondrial accumulation is not only considered a hallmark of IPF pathology but also a key driver of disease progression. Recent evidence highlights the impact of disrupted mitochondrial function in alveolar type II epithelial cells (AEC IIs), fibroblasts, and alveolar macrophages (AMs) on the pathogenesis of IPF(Yu et al. 2018; McDonough et al. 2019). Compared to fibroblasts from healthy individuals, IPF lung fibroblasts exhibit reduced mitochondrial mass, impaired membrane structures, and altered cristae morphology, indicating changes in mitochondrial function (McDonough et al. 2019). Mitochondrial dynamics regulate mitochondrial morphology and function through fusion and fission, playing a vital role in maintaining cellular homeostasis, adapting to stress, and controlling cell fate(Yang et al. 2023; Chan 2020; Quintana-Cabrera et al. 2023). Key markers include mitofusin protein 1(MFN1), mitofusin protein 2(MFN2), and optic atrophy 1(OPA1), while fission is marked by dynamin-related protein 1(DRP1) (Giacomello et al. 2020; Wai et al. 2016; Chen et al. 2023). Additionally, ACSS3 alleviates pulmonary fibrosis by promoting mitochondrial biogenesis in AEC IIs, stabilizing mitochondrial dynamics, and improving metabolic homeostasis(Wang et al. 2024). Conditional deletion of Mfn1 leads to mitochondrial fragmentation, while deletion of Mfn2 causes mitochondrial swelling after bleomycin exposure(Habermann et al. 2020). Furthermore, mice lacking both Mfn1 and Mfn2 in AEC IIs spontaneously develop severe mitochondrial dysfunction and pulmonary fibrosis(Chung et al. 2019). These findings suggest that targeting mitochondria-related proteins may provide new therapeutic strategies for treating pulmonary fibrosis.

Calcium/calmodulin dependent protein kinase 2 (CAMKK2) is, a serine/threonine protein kinase, is involved in various physiological processes, including autophagy, mitochondrial homeostasis, energy metabolism. CAMKK2 is regulated by NEDD4L in diabetic nephropathy, affecting mitochondrial dynamics and ROS production(Han et al. 2024). Diosgenin targets CAMKK2 to improve autophagy and mitochondrial dynamics in diabetic nephropathy(Zhong et al. 2023). Aβ42 oligomers inhibit synaptic loss by coordinating mitochondrial fission and mitophagy through the CAMKK2-AMPK dependent pathway(Lee et al. 2022). To summarize, CAMKK2 plays a crucial role in regulating mitochondrial function. Although downregulation of CAMKK2 has been observed in the pathological progression of pulmonary fibrosis, its underlying mechanisms are still unclear. Therefore, our study aims to elucidate the role of CAMKK2 and relations with disrupted mitochondrial dynamics function in fibroblasts, as well as to evaluate its potential as a therapeutic target for IPF.

## Results

### The alterations of CAMKK2 expression and mitochondrial dynamics in fibrotic lungs and TGF-β1 stimulated fibroblasts

**Fig. 1:**
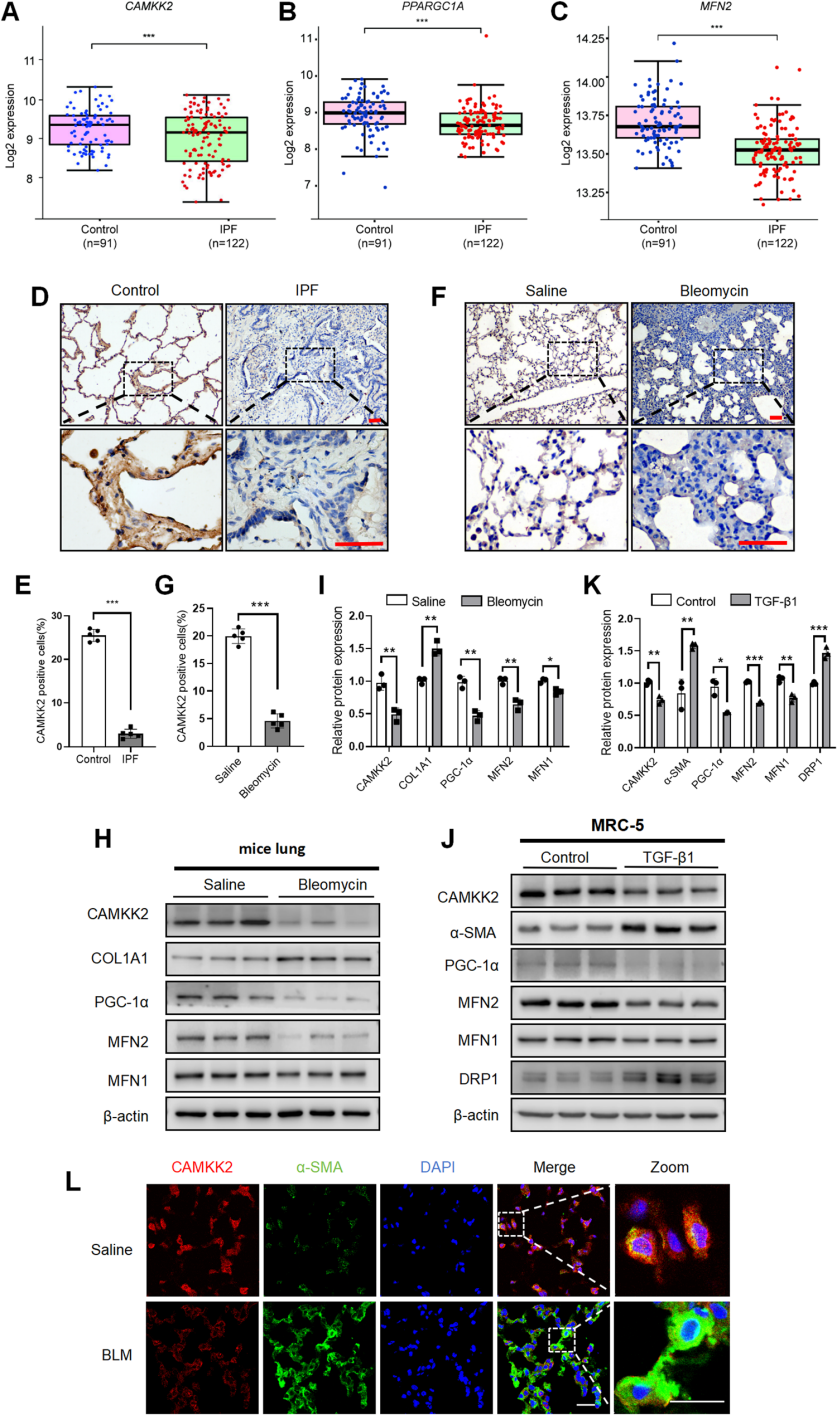
The alterations of CAMKK2 expression and mitochondrial dynamics in fibrotic lungs and TGF-β1 stimulated fibroblasts. **A-C** Re-analysis of the *CAMKK2*,* PPARGC1A* and *MFN2* in the GSE47460 microarray datasets. **D**-**E** Representative images and quantification of IHC staining for CAMKK2 in lung sections from IPF patients and non-IPF control (*n* = 5), Scale bars = 50 μm. **F**-**G** Representative images and quantification of IHC staining for CAMKK2 in lung sections from bleomycin induced mice and saline control (*n* = 5), Scale bars = 50 μm. **H**-**I** Western blotting analysis and quantification of CAMKK2, COL1A1, PGC-1α, MFN2 and MFN1 levels in mouse lung homogenates (*n* = 3). **J**-**K** Western blotting analysis and quantification of CAMKK2, α-SMA, PGC-1α, MFN2, MFN1 and DRP1 levels in MRC-5 cells treated for 48 h with and without TGF-β1 (5 ng/mL) (*n* = 3). **L** Representative images of co-staining of CAMKK2 with α-SMA (a fibroblast marker) in lung tissues from bleomycin induced mice and saline control (*n* = 5). Scale bars = 50 μm. The data represent one of at least three independent experiments. Data are shown as the mean ± SD. *P < 0.05; ** <0.01; ***P < 0.001; ns = not significant

Dysfunctional mitochondrial accumulation is not only considered a hallmark of IPF pathology but also a key driver of disease progression. The expression levels of CAMKK2, along with PGC-1α (mitochondrial biogenesis maker), MFN1 and MFN2 (mitochondrial dynamics makers) were examined in fibrotic lung tissue. A reanalysis of the GSE47460 datasets demonstrated a significant downregulation in the mRNA expression levels of *CAMKK2*,* PPARGC1A* and *MFN2* in the lung tissues of IPF (Fig. 1A-C), while *MFN1* showed a less pronounced downregulation (Fig. S1A). IHC analysis demonstrated that disease-associated CAMKK2 downregulation in IPF lungs compared to non-IPF controls (Fig. 1D-E).

A murine model of pulmonary fibrosis was developed through the administration of bleomycin to facilitate subsequent research. IHC analysis revealed that CAMKK2 expression was downregulated in fibrotic regions of bleomycin-treated mice compared to controls (Fig. 1F-G). Western blotting and RT-qPCR analysis demonstrated that CAMKK2 expression was downregulated at both protein (Fig. 1H-I) and mRNA levels (Fig. S1B) in lung tissues from bleomycin-treated mice. Co-staining results demonstrated that CAMKK2 mainly co-localized in α-SMA^+^ fibroblasts (Fig. 1L), with minimal detection in SPC^**+**^ epithelial cells in mouse lung tissues (Fig. S1C). Notably, CAMKK2 expression was significantly reduced in bleomycin-induced fibrotic lungs compared to saline group in co-staining views. Furthermore, a significant downregulation of PGC-1α, MFN2 and MFN1 was observed in the bleomycin induced mice lung, following with a marked upregulation of the fibrotic gene COL1A1 (Fig. 1H- I).

TGF-β1 stimulation can induces fibroblasts differentiation into myofibroblasts, characterized by an upregulation of the fibrotic marker α-SMA in MRC-5 cells (Fig. 1J-K). Western blotting analysis demonstrated that the expression of CAMKK2, PGC-1α, MFN2 and MFN1 was reduced, accompanied by an upregulation of DRP1 in MRC-5 cells stimulated by TGF-β1(Fig. 1J-K). The results suggested notable alterations of CAMKK2 expression with disrupted mitochondrial dynamics may be pivotal to pulmonary fibrosis.

### Inhibition of CAMKK2 enhanced fibroblast activation and ECM production

**Fig. 2:**
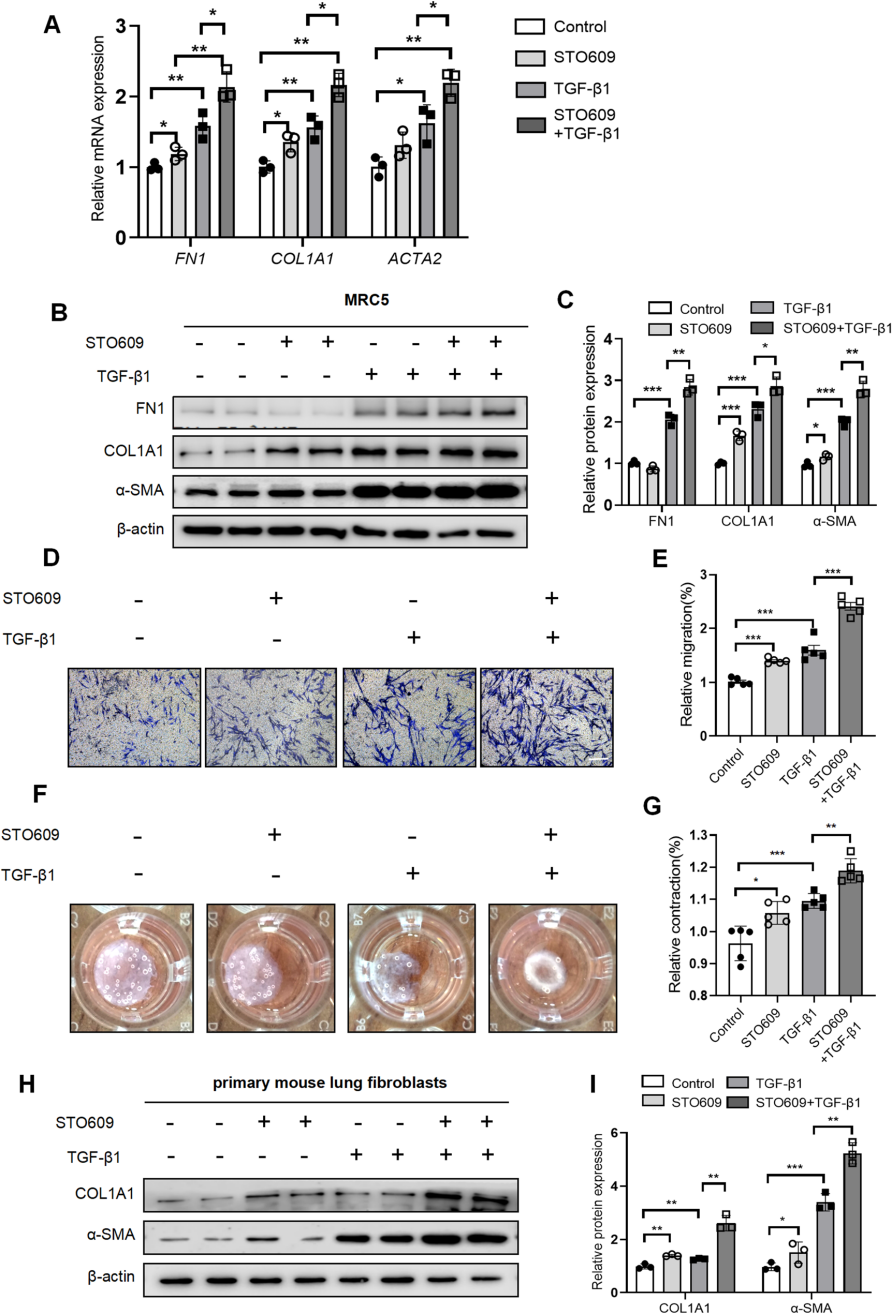
Inhibition of CAMKK2 enhanced fibroblast activation and ECM production. MRC-5 cells or primary mouse lung fibroblasts were pretreated with STO-609 (a selective CAMKK2 inhibitor, 10µM) or empty control for 24 h, then stimulated with and without TGF-β1 (5 ng/mL) for 24 h before sample collection. **A** mRNA expression of *FN1*, *COL1A1* and *ACTA2* in MRC-5 cells (*n* = 3). **B**-**C** Western blotting analysis and quantification of FN1, COL1A1 and α-SMA in MRC-5 cells (*n* = 3). **D**-**E** Representative images and quantification of migration activity by transwell assay in MRC-5 cells (*n* = 5). Scale bars = 100 μm. **F**-**J** Representative images and quantification of contraction activity by collagen gel contraction assay in MRC-5 cells (*n* = 5). **H**-**I** Western blotting analysis and quantification of COL1A and α-SMA in primary mouse lung fibroblasts (*n* = 3). The data represent one of at least three independent experiments. Data are shown as the mean ± SD. *P < 0.05, **P < 0.01, ***P < 0.001; ns = not significant

To further elucidate the role of CAMKK2 downregulation in fibroblasts, we examined the impact of CAMKK2 inhibition in MRC-5 cells and primary mouse lung fibroblasts stimulated by TGF-β1 in vitro. Compared to the TGF-β1 treatment group, STO-609 (a selective CAMKK2 inhibitor) treatment further enhanced the expression of fibrotic makers (FN1, COL1A1 and α-SMA) at both the mRNA and protein levels in TGF-β1 induced MRC-5 cells (Fig. 2A-C). The transwell assay analysis demonstrated that inhibition of CAMKK2 significantly augmented the migration activity in MRC-5 fibroblasts, irrespective of TGF-β1 stimulation status (Fig. 2D-E). Similarly, inhibition of CAMKK2 significantly promoted the contraction activity in MRC-5 cells, irrespective of TGF-β1 stimulation status (Fig. 2F-G). Additionally, inhibition of CAMKK2 upregulated the expression of COL1A1 and α-SMA at the protein levels in primary mouse lung fibroblasts with or without TGF-β1 treatment (Fig. 2H-I). As STO-609 demonstrates selectivity for the CAMKK family without exclusive specificity for CAMKK2, it is necessary to validate the observed effects through CAMKK2-specific knockdown experiments to confirm target specificity. We did perform sh-CAMKK2 knockdown experiments (50–60% efficiency), showing significant upregulation of fibrotic markers expression (FN1, COL1A1 and α-SMA) in the MRC-5 cells (Fig. S2 A-B), which consistently reproduced the inhibitor’s effects on fibrotic genes expression. In summary, these findings implied that inhibition of CAMKK2 enhanced fibroblast activation and ECM production.

###  Overexpression of CAMKK2 suppressed fibroblast activation and ECM production in TGF-β1-stimulated fibroblasts

**Fig. 3:**
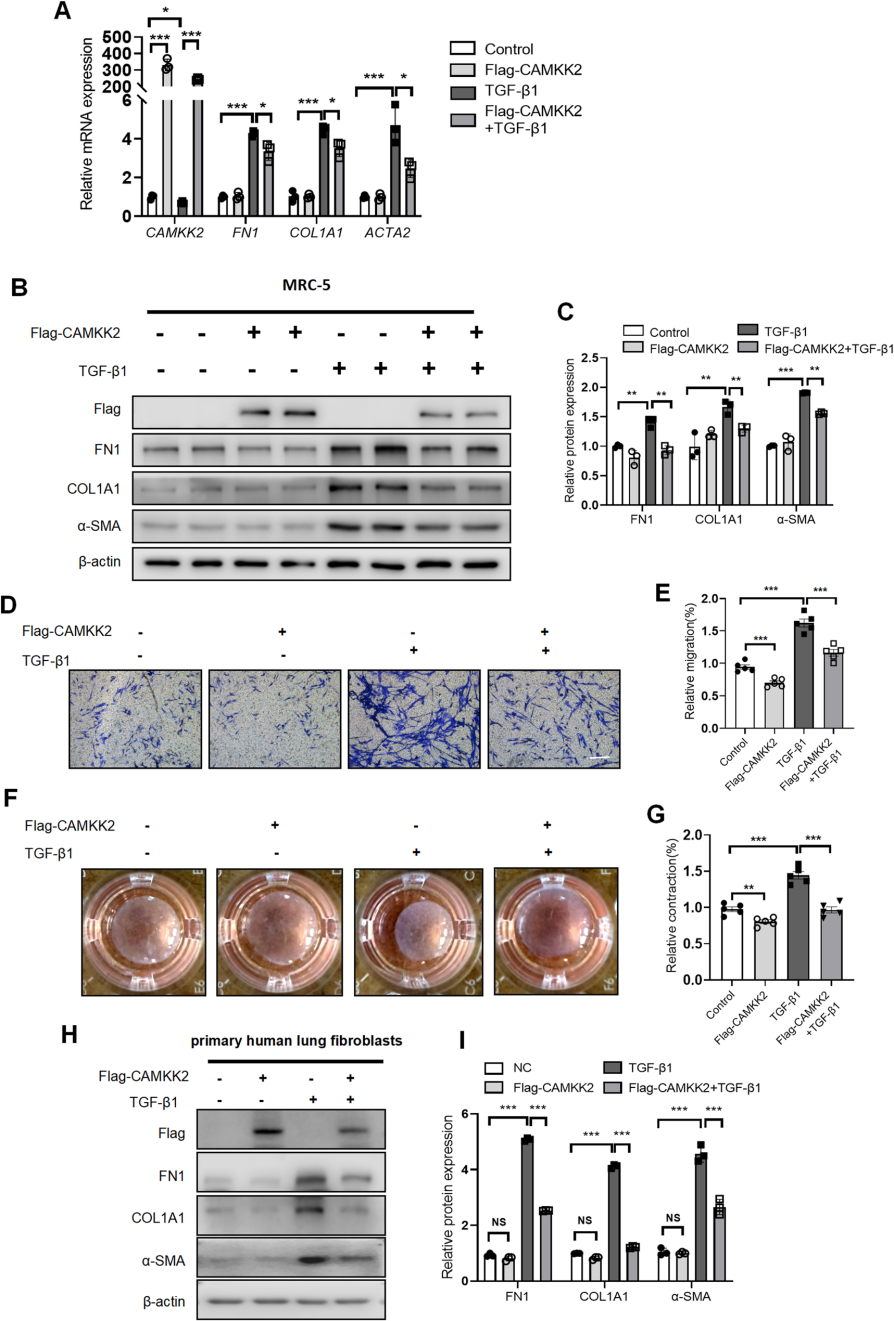
Overexpression of CAMKK2 suppressed fibroblast activation and ECM production in TGF-β1-stimulated fibroblasts. MRC-5 cells or primary human lung fibroblasts were transfected with pcDNA3.1 plasmid or pcDNA3.1-Flag-CAMKK2 plasmid for 24 h, then stimulated with and without TGF-β1 (5 ng/mL) for 24 h before sample collection. **A** mRNA expression of *CAMKK2*, *FN1*,* COL1A1* and *ACTA2* in MRC-5 cells (*n* = 3). **B**-**C** Western blotting analysis and quantification of Flag-CAMKK2, FN1, COL1A1 and α-SMA in MRC-5 cells (*n* = 3). **D**-**E** Representative images and quantification of migration activity by transwell assay in MRC-5 cells (*n* = 5). Scale bars = 100 μm. **F**-**G** Representative images and quantification of contraction activity by collagen gel contraction assay in MRC-5 cells (*n* = 5). **H**-**I** Western blotting analysis and quantification of Flag-CAMKK2, FN1, COL1A1 and α-SMA levels in the primary human lung fibroblasts. The data represent one of at least three independent experiments. Data are shown as the mean ± SD. *P < 0.05, **P < 0.01, ***P < 0.001. ns = not significant

Conversely, we evaluate the impact of overexpressed CAMKK2 in MRC-5 cells and primary human lung fibroblasts stimulated by TGF-β1. Initially, the expression levels of CAMKK2 in MRC-5 cells were assessed at both the RNA and protein levels to confirm the successful construction of CAMKK2 overexpressing (Flag-CAMKK2) cells (Fig. 3A-C). Compared to the TGF-β1 treatment group, overexpression of CAMKK2 significantly downregulated the expression of FN1, COL1A1 and α-SMA in the Flag-CAMKK2 + TGF-β1 stimulated MRC-5 cells (Fig. 3A-C). Additionally, TGF-β1 stimulation increased fibroblast migration activity, but these effects were partially reversed by CAMKK2 overexpression in TGF-β1 stimulated MRC-5 cells by transwell assay (Fig. 3D-E) and wound-healing assay (Fig. S3 A-B). Similarly, CAMKK2 overexpression inhibited fibroblasts contraction activity in MRC-5 cells (Fig. 3F-G). Additionally, overexpression of CAMKK2 unregulated the expression of COL1A1 and α-SMA at the protein levels in primary human lung fibroblasts stimulated by TGF-β1 (Fig. 3H-I). Collectively, these findings proposed that CAMKK2 overexpression suppressed fibroblast activation and ECM production in TGF-β1-stimulated lung fibroblasts.

### CAMKK2 modulated mitochondrial dynamics in TGF-β1-stimulated fibroblasts

**Fig. 4:**
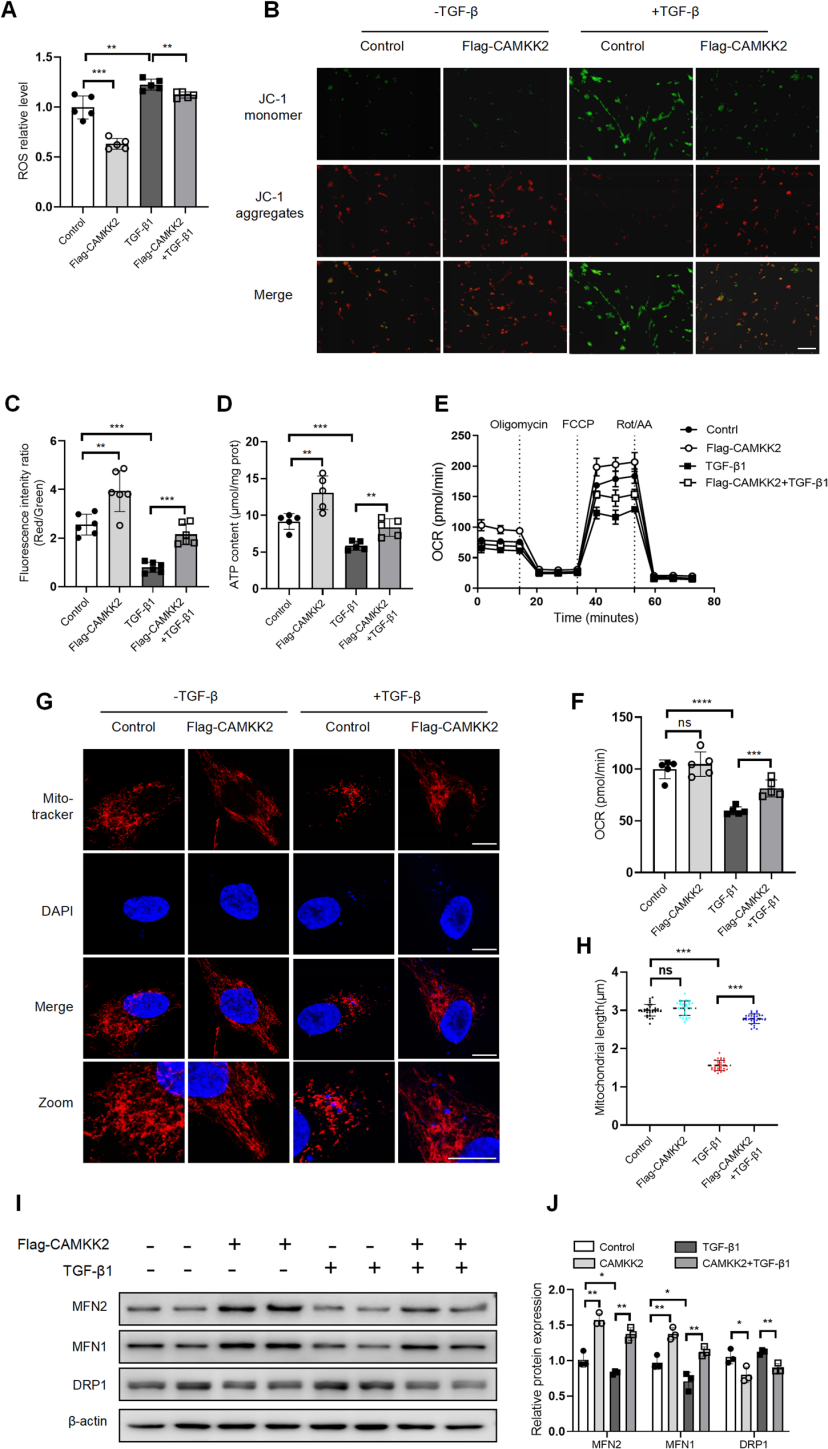
CAMKK2 modulated mitochondrial dynamics in TGF-β1-stimulated fibroblasts. MRC-5 cells were transfected with pcDNA3.1 plasmid or pcDNA3.1-Flag-CAMKK2 plasmid for 24 h, then stimulated with and without TGF-β1 (5 ng/mL) for 24 h before sample collection. **A** ROS relative level of MRC-5 cells (*n* = 5). **B** Representative images of MMP by JC-1staining in MRC-5 cells, Scale bars = 100 μm. **C** Quantification analysis of fluorescence intensity ratio (Red/Green) of MMP (*n* = 6). **D** ATP content (µmol/mg prot) of MRC-5 cells(*n* = 5). **E**-**F** The OCR (pmol/min) of MRC-5 cells by seahorse assay(*n* = 5). **G** Representative images of Mito-tracker (red) staining in MRC-5 cells, Scale bars = 50 μm. **H** Metrics of mitochondria length (*n* = 30 for each group). **I**-**J** Western blotting analysis and quantification of MFN2, MFN1 and DRP1 in MRC-5 cells (*n* = 3). The data represent one of at least three independent experiments. Data are shown as the mean ± SD. *P < 0.05, **P < 0.01, ***P < 0.001. ns = not significant

To further investigate the impact of CAMKK2 on mitochondrial function, mitochondrial function indicators (ROS level, mitochondrial membrane potential (MMP), ATP synthesis and oxygen consumption rate (OCR) were assessed. In vitro, CAMKK2 overexpression significantly reduced the ROS levels in MRC-5 cells (Fig. 4A). The red-to-green fluorescence intensity ratio was quantitatively analyzed to assess changes in MMP by JC-1 staining. Fluorescence imaging and quantitative analysis of the JC-1 fluorescence ratio (Red/Green) demonstrated that CAMKK2 overexpression effectively attenuated the TGF-β1-induced reduction in MMP of MRC-5 cells (Fig. 4B–C). ATP synthesis content and OCR are employed as metrics to evaluate the extent of mitochondrial energy metabolism. Compared to the TGF-β1 treatment group, CAMKK2 overexpression elevated the ATP content (Fig. 4D) and maximal respiratory capacity in the MRC-5 stimulated by TGF-β1(Fig. 4E-F).

Mitochondrial morphology/structure, visualized using Mito-tracker red staining, showed that TGF-β1-induced increase in fragmented mitochondria in MRC-5 cells. However, CAMKK2 overexpression particularly normalized the mitochondrial network morphology/structure (Fig. 4G-H). The mitochondrial length was used to represent the mitochondrial network morphology. The less mitochondrial networks, the shorter mitochondrial length. Aberrant mitochondrial fragmentation is often correlated with mitochondrial dynamics homeostasis. Western blotting analysis demonstrated that CAMKK2 overexpression also restored TGF-β1-induced dysfunctional mitochondrial dynamics by promoting mitochondrial fusion protein (MFN1 and MFN2) expression and reducing mitochondrial fission protein (DRP1) in MRC-5 cells (Fig. 4I-J). Collectively, these findings demonstrated that CAMKK2 overexpression modulated mitochondrial dynamics in TGF-β1-stimulated MRC-5 cells.

### CAMKK2 modulated mitochondrial dynamics homeostasis via the AMPK/PGC-1α signaling pathway

**Fig. 5:**
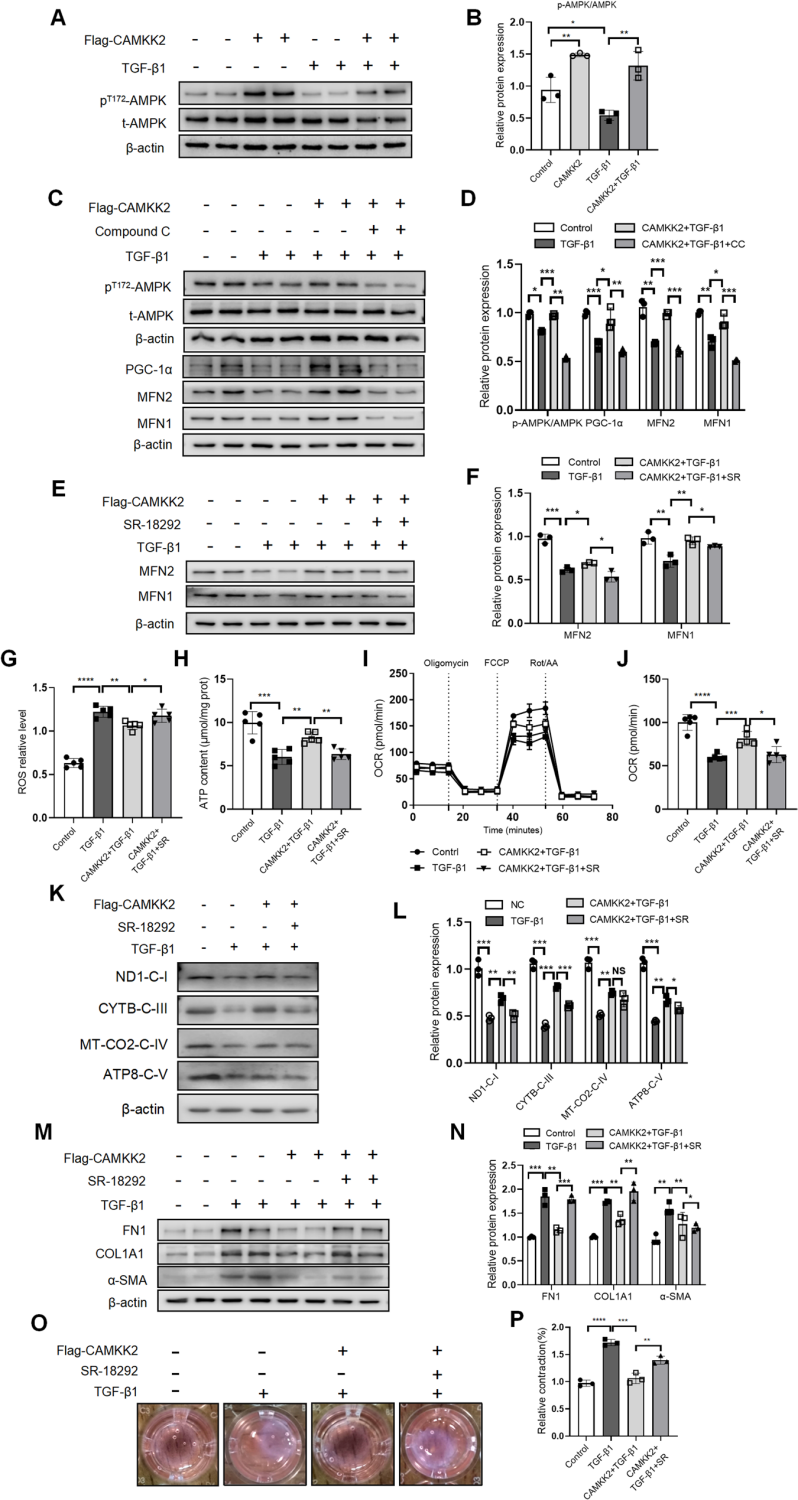
CAMKK2 modulated mitochondrial dynamics homeotasis via the AMPK/PGC-1α signaling pathway. **A**-**B** Western blotting analysis and quantification of p^T172^-AMPKα and t-AMPKα in MRC-5 cells transfected with pcDNA3.1 plasmid or pcDNA3.1-Flag-CAMKK2 plasmid for 24 h, then stimulated with TGF-β1 (5 ng/mL) for 24 h (*n* = 3). **C**-**D** Western blotting analysis and quantification of p^T172^-AMPKα, t-AMPKα, PGC-1α, MFN2 and MFN1 in MRC-5 cells transfected with pcDNA3.1 plasmid or pcDNA3.1-Flag-CAMKK2 plasmid for 24 h, followed with pre-treatment with Compound C (a potent AMPK inhibitor, 10µM) for 2 h, before stimulation with 5 ng/mL TGF-β1(*n* = 3). **E**-**F** Western blotting analysis and quantification of MFN2 and MFN1 in MRC-5 cells transfected with pcDNA3.1 plasmid or pcDNA3.1-Flag-CAMKK2 plasmid for 24 h, followed with pre-treatment with SR-18,292 (a specific PGC-1α inhibitor, 20µM) for 2 h, before stimulation with 5 ng/mL TGF-β1(*n* = 3), as below. **G** ROS relative level of MRC-5 cells (*n* = 5). **H** ATP content (µmol/mg prot) of MRC-5 cells(*n* = 5). **I**-**J** The OCR (pmol/min) of MRC-5 cells (*n* = 5). **K**-**L** Western blotting analysis and quantification of ND1-C-I, CYTB-C-III, MT-CO2-C-IV and ATP8-C-V in MRC-5 cells (*n* = 3). **M**-**N** Western blotting analysis and quantification of FN1, COL1A1 and α-SMA in MRC-5 cells(*n* = 3). **O**-**P** Representative images and quantification of contraction activity by collagen gel contraction assay in MRC-5 cells (*n* = 3). The data represent one of at least three independent experiments. Data are shown as the mean ± SD. *P < 0.05, **P < 0.01, ***P < 0.001. ns = not significant

To further investigate mechanism of CAMKK2 modulated mitochondrial dynamics homeostasis, downstream signaling pathways were examined. Novelty, CAMKK2 activated AMPK through phosphorylation at threonine 172 in MRC-5 cells, regardless of TGF-β1 stimulation (Fig. 5A-B). Inhibition of CAMKK2 further suppressed AMPK phosphorylation in MRC-5 cells (Fig. S4 A-B). To further explore the relationship between AMPK signaling pathway and mitochondrial dynamics, Compound C (a potent AMPK inhibitor) was used to detect changes of mitochondrial dynamics in rescue experiments. Compound C inhibitor was reduced p-AMPK expression in MRC-5 cells (Fig. 5C-D). Additionally, CAMKK2 overexpression markedly enhanced the expression of MFN2, MFN1 and PGC-1α, while a partially reversed this upregulation by Compound C in MRC-5 cells (Fig. 5C-D).

Studies have shown that mitochondrial biogenesis can regulate mitochondrial fusion(Bhatia et al. 2022). We further explored the relationship between PGC-1α and mitochondrial fusion in MRC-5 fibroblasts. SR-18,292 (a specific PGC-1α inhibitor) was used to detect changes of mitochondrial dynamics in rescue experiments. Interestingly, compared to Flag-CAMKK2 + TGF-β1 group, the expression of mitochondrial fusion protein (MFN1 and MFN2) was significantly decreased in MRC-5 cells with SR-18,292 (Fig. 5E-F). Meanwhile, compared to Flag-CAMKK2 + TGF-β1 group, SR-18,292 treatment induced ROS levels increasing (Fig. 5G), ATP content decreasing (Fig. 5H), and maximal respiratory capacity decreasing (Fig. 5I-J) in MRC-5 cells. Next, we systematically analyzed the expression profiles of oxidative phosphorylation (OXPHOS) pathway components, given their established regulation by PGC-1α. Western blotting analysis demonstrated that TGF-β1 treatment significantly downregulated core OXPHOS complexes (ND1-C-I, CYTB-C-III, MT-CO2-C-IV, ATP8-C-V; *p* < 0.001) expression, while CAMKK2 overexpression partially but significantly rescued this suppression (*p* < 0.01 vs. TGF-β1 alone). Importantly, this rescue effect was partially abolished by SR-18,292 (Fig. 5K-L). Furthermore, Western blotting analysis demonstrated that SR-18,292 partially reversed the expression of FN1, COL1A and α-SMA in MRC-5 cells, compared to Flag-CAMKK2 + TGF-β1 group (Fig. 5M-N). Fibroblast contraction activity notably increased treated with SR-18,292 compared to Flag-CAMKK2 + TGF-β1 treated MRC-5 cells group (Fig. 5O-P). Collectively, these findings suggested that CAMKK2 modulated mitochondrial dynamics and OXPHOS function via the AMPK/PGC-1α signaling pathway in TGF-β1-stimulated MRC-5 cells.

### Camkk2 attenuated bleomycin induced pulmonary fibrosis in mice

**Fig. 6:**
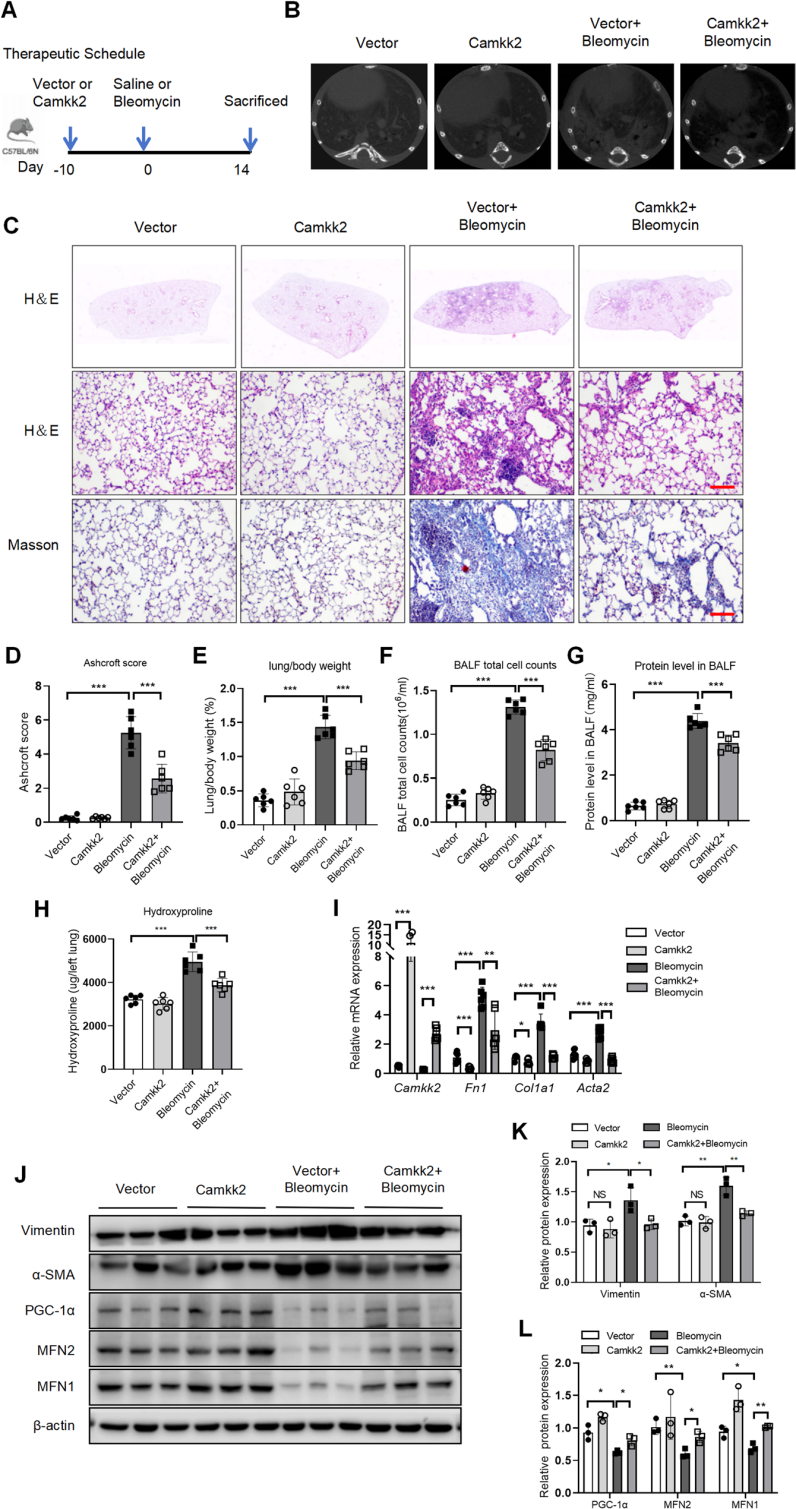
Camkk2 attenuated bleomycin induced pulmonary fibrosis in mice. C57BL/6 N mice were treated with AAV6, AAV6-Camkk2-Flag, and bleomycin challenge in mice, as described in methods. **A** The schematic diagram depicts the timeline of the experiment. **B** Representative micro-CT images of mouse lungs on the day 14 after bleomycin challenge. **C** Representative images of H&E and Masson trichrome staining of lung sections. Scale bars = 100 μm. **D** Ashcroft score of mice(*n* = 6). **E** Lung/body weight of mice (*n* = 6). **F** BALF total cell counts of mice (*n* = 6). **G** Protein level in BALF of mice (*n* = 6). **H** Hydroxyproline content in the left lung (*n* = 6). **I** mRNA expression of *Camkk2*, *Fn1*, *Col1a1*, and *Acta2* in mouse lung homogenates assessed by qRT-PCR (*n* = 6). **J**-**L** Western blotting analysis and quantification of Vimentin, α-SMA, PGC-1α, MFN2 and MFN1 levels in mouse lung homogenates (*n* = 3). The data represent one of at least three independent experiments. Data are shown as the mean ± SD. *P < 0.05, **P < 0.01, ***P < 0.001. ns = not significant

To evaluate the therapeutic efficacy of CAMKK2 in pulmonary fibrosis, we employed adeno-associated virus serotype AAV6 to deliver either pcAAV-CMV-Camkk2-Flag or pcAAV-CMV to C57BL/6 N mice through intratracheal administration. Subsequently, ten days later, the mice received intratracheal administration of either bleomycin or saline (Fig. 6A). Micro-CT imaging demonstrated pronounced fibrosis in the lung tissues of mice subjected to Vector + Bleomycin (pcAAV-CMV + Bleomycin) challenge, compared to Vector (pcAAV-CMV + Saline) group. However, compared to Vector + Bleomycin group, the group treated with Camkk2 + Bleomycin (pcAAV-CMV-Camkk2-Flag + Bleomycin) exhibited a reduction in lung tissue damage (Fig. 6B). H&E and Masson staining confirmed that Camkk2 + Bleomycin group exhibited a significant attenuation of pulmonary injury and a reduction in collagen deposition within the lung tissue, in comparison to the group treated with Vector + Bleomycin (Fig. 6C). Properly, the Camkk2 + Bleomycin group exhibited significantly lower Ashcroft scores compared to the Vector + Bleomycin group (Fig. 6D). Notably, compared with Vector group, we observed an increase in lung/body weight ratios (Fig. 6E), BALF total inflammatory cells counts (Fig. 6F), protein levels in BALF (Fig. 6G), and hydroxyproline content (Fig. 6H) in Vector + Bleomycin group. However, Camkk2 + Bleomycin group mice resulted in a reduction in lung/body weight ratios, BALF total inflammatory cells counts, protein levels in BALF, and hydroxyproline content (Fig. 6E-H), compared to Vector + Bleomycin group.

Camkk2 expression was found to be elevated through qRT-PCR, thereby proving the successful establishment of the Camkk2 overexpressed efficiency in mice (Fig. 6I). Moreover, qRT-PCR analysis also showed a significant decrease in fibrosis markers such as *Fn1*,* Col1a1* and *Acta2* in the lungs of the Camkk2 + Bleomycin group mice, compared to Vector + Bleomycin group (Fig. 6I). Western blotting results demonstrated fibrosis markers (Vimentin and α-SMA) were also markedly reduced in Camkk2 + Bleomycin group mice, compared to Vector + Bleomycin group (Fig. 6J-K). Additionally, the changes of mitochondrial dynamics indicators were verified after Camkk2 overexpression in vivo. Bleomycin markedly reduced the protein expression levels of PGC-1α, MFN2, and MFN1 in the lung tissue. In contrast, the administration of Camkk2 + Bleomycin resulted in an increase in the levels of PGC-1α, MFN2, and MFN1 in mice, compared to Vector + Bleomycin group (Fig. 6J, L). Collectively, these results implied that AAV-Camkk2 attenuated bleomycin induced pulmonary fibrosis in mice.

## Discussion

IPF, a chronic lung disease of unknown etiology, is defined by progressive interstitial fibrosis, which is characterized by abnormal fibroblast activity and ECM remodeling(Bonella et al. 2023; Maher 2024). Lung fibroblasts from IPF patients display reduced mitochondrial mass, compromised membrane integrity, aberrant cristae morphology, and dysregulated mitochondrial dynamics(McDonough et al. 2019; Pokharel et al. 2024). Collectively, these alterations establish mitochondrial dysfunction as a hallmark feature of IPF pathogenesis. Dysregulation of mitochondrial dynamic processes has been implicated in the pathogenesis of numerous diseases(Yu et al. 2018; Chan 2020; Piamsiri et al. 2024), highlighting their critical importance in physiological and pathological contexts. Thus, targeting mitochondria-related proteins could offer innovative therapeutic strategies for pulmonary fibrosis. In the present study, a reanalysis of the GSE47460 datasets revealed significantly decreased expressions of *PPARGC1A* and *MFN2* in the IPF lung tissues. Moreover, there was a pronounced reduction in the levels of MFN2 and MFN1 in the bleomycin-induced fibrotic mouse model and TGF-β1-stimulated MRC-5 cells. Elucidating the regulatory relationship between mitochondrial dynamics and fibroblast differentiation is critical for understanding pulmonary fibrosis pathogenesis.

CAMKK2 plays a crucial role in physiological processes such as autophagy(Jing et al. 2021), mitochondrial function (Han et al. 2024; Sabbir et al. 2021), and energy metabolism(Lee et al. 2022)^[19]^. Abeta42 oligomers trigger synaptic loss through CAMKK2-AMPK-dependent effectors coordinating mitochondrial fission and mitophagy(Lee et al. 2022). However, the role of CAKK2 in pulmonary fibrosis remains unclear. CAMKK2 expression was notably reduced in human and mice fibrotic lungs, and TGF-β1-stimulated MRC-5 cells. Moreover, AAV-Camkk2 has been shown to alleviate bleomycin-induced pulmonary fibrosis in mice. Therefore, investigating the mechanism of CAMKK2 in pulmonary fibrosis holds significant importance. The hallmark features of IPF include abnormal fibroblasts activation, myofibroblast differentiation and excessive ECM deposition(Adegunsoye et al. 2024; Karampitsakos et al. 2023). Activation of TGF-β1 signaling is a common characteristic of fibrotic conditions(Lan 2011). Inhibition of CAMKK2 by STO609(a selective CAMKK2 inhibitor) and sh-CAMKK2 enhanced the expression of α-SMA (fibroblast differentiation maker), FN1 and COL1A1 (ECM makers) in TGF-β1-stimulated MRC-5 cells. Conversely, CAMKK2 overexpression suppressed the expression of α-SMA, FN1 and COL1A1 in TGF-β1-stimulated MRC-5 cells and primary human lung fibroblasts. Taken together, these data collectively suggested that CAMKK2 inhibited TGF-β1-stimulated lung fibroblasts activation and ECM production. Meanwhile, CAMKK2 overexpression effectively reduced ROS levels, enhanced MMP, ATP synthesis content and respiratory capacity in TGF-β1-stimulated MRC-5 cells. Additionally, CAMKK2 overexpression elevated the expression of MFN1 and MFN2, while reducing DRP1 expression. Taken together, these data collectively suggested that CAMKK2 overexpression inhibited fibroblast activation and ECM production through modulating mitochondrial function in TGF-β1-stimulated MRC-5 cells.

Mitochondrial biogenesis, a process involving both mitochondrial and nuclear genes, increases mitochondrial size and mass(Pfanner et al. 2019). Disrupted mitochondrial biogenesis can lead to elevated ROS and contribute to pulmonary fibrosis(Gu et al. 2019; Bueno et al. 2020). Peroxisome proliferator-activated receptor γ coactivator 1α (PGC-1α) is a pivotal transcriptional coactivator that plays a central role in processes such as energy metabolism(Qin et al. 2020) and mitochondrial biogenesis(Abu Shelbayeh et al. 2023; Nam et al. 2022). Thyroid hormone treatment improves mitochondrial function via PGC-1α in AEC II(Yu et al. 2018). Inhibiting PGC-1α promotes myofibroblast differentiation in lung fibroblasts. In this study, we demonstrated that significant downregulation of PGC-1α expression was observed in the IPF datasets GSE47460, bleomycin-induced mouse lungs, and TGF-β1-stimulated MRC-5 cells. Overexpression of CAMKK2 upregulated the expression of p^T172^AMPK and PGC-1α in MRC-5 cells. However, these effects were abrogated by Compound C (a potent AMPK inhibitor). Specifically, Compound C partially reversed the upregulation of mitochondrial fusion protein (MFN1 and MFN2) induced by CAMKK2 overexpression in TGF-β1-stimulated MRC-5 cells. Interestingly, further studies revealed that treatment with SR-18,292, a specific inhibitor of PGC-1α, significantly decreased mitochondrial fusion protein (MFN1 and MFN2) and OXPHOS complexes (ND1-C-I, CYTB-C-III, ATP8-C-V) expression in TGF-β1-stimulated MRC-5 cells with CAMKK2 overexpression. To summary, these data collectively suggest that CAMKK2 modulated mitochondrial dynamics and OXPHOS function via the AMPK/PGC-1α signaling pathway in TGF-β1-stimulated MRC-5 cells.

Despite the notable advantages of our study, several limitations must be acknowledged. A major limitation of this study is the use of MRC-5 cells as a myofibroblast differentiation for IPF. While MRC-5 cells provide a standardized model for studying myofibroblast differentiation mechanisms, we recognize they represent embryonic fibroblasts and may not fully recapitulate the aging-related pathophysiology of IPF. The use of primary human lung fibroblasts derived from IPF patients would provide a more representative model that better reflects the disease-specific characteristics. Another limitation of this study is its sole focus on exploring the functions of CAMKK2 in lung fibroblasts. This restricted scope means that the study does not examine CAMKK2’s roles in epithelial cells and macrophages, thus precluding a comprehensive understanding of its functions across different cell types. Additionally, further research using cell type-specific, conditional knockout mice is needed to clarify the cell type-specific effects of CAMKK2 in fibrotic lung tissue.

**Fig. 7:**
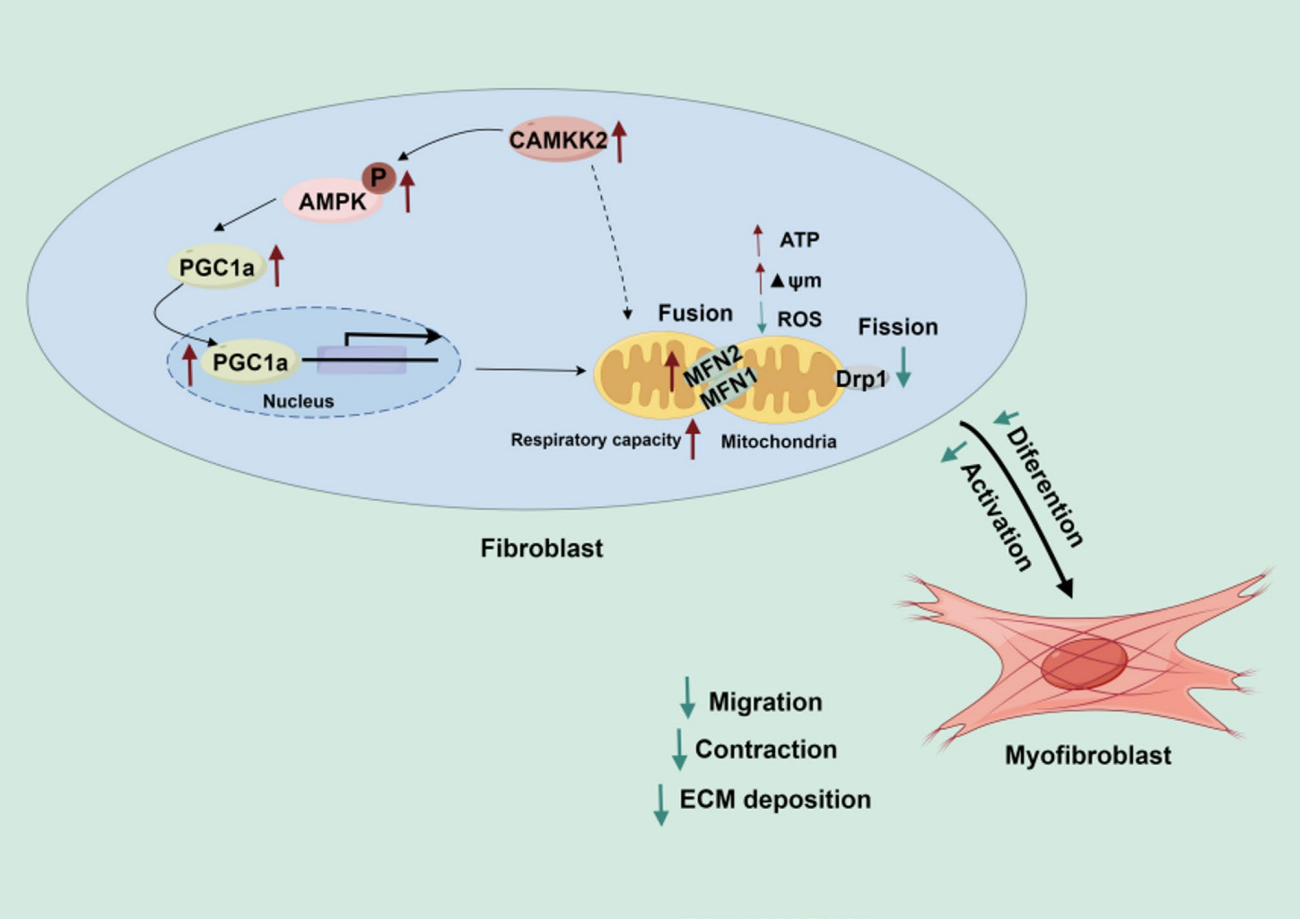
Schematic overview of mechanism (By Figdraw). The proposed model shows that CAMKK2 overexpression inhibits fibroblasts activation and myofibroblasts differentiation by reinstating mitochondrial dynamics via the AMPK/PGC-1α signaling pathway to alleviate pulmonary fibrosis

In summary, this study collectively investigated that CAMKK2 overexpression inhibits fibroblast activation and ECM production by regulating mitochondrial dynamics and OXPHOS function via AMPK/PGC-1α signaling pathway to alleviate pulmonary fibrosis (Fig. 7). Therefore, targeting CAMKK2 presents a novel and promising therapeutic strategy for the treatment of pulmonary fibrosis.

## Materials and methods

### Clinical samples

Lung specimens from IPF patients were obtained through lung biopsy, while lung tissues of the control group were collected from the non-diseased marginal areas of lung cancer specimens, providing a suitable reference for comparison with IPF samples. IPF and control non-IPF lung was diagnosed according to consensus criteria based on ATS/ERS/JRS/ALAT Clinical Practice Guidelines(Raghu et al. 2018). Informed consent was meticulously secured from every patient prior to any surgical operations.

### Bioinformatics analysis

Transcriptomic microarray data and clinical metadata from the GSE47460 cohort were retrieved from the Gene Expression Omnibus (GEO) database (https://www.ncbi.nlm.nih.gov/geo/). To mitigate platform-specific batch effects, we exclusively analyzed raw data generated from the GPL14550 platform (Agilent-028282), comprising 91 healthy controls (normal pulmonary function) and 122 IPF cases (UIP subtype confirmed per 2018 ATS criteria). To maintain analytical objectivity, we preserved the original normalization pipeline without additional preprocessing. Gene annotation was performed with the Nanoprobe R package (v0.3.6), employing a sequence alignment-based approach. *CAMKK2*,* PPARGC1A*, *MFN2* and *MFN1* expression was compared between IPF and controls using Wilcoxon rank-sum test.

### Reagents and cell culture

Recombinant human transforming growth factor (TGF)-β1 (240-B) was procured from R&D Systems (Minneapolis, MN). A selective CAMKK2 inhibitor STO-609(HY-19805), a potent AMPK inhibitor Compound C (dorsomorphin dihydrochloride, HY-13418 A), a specific PGC-1α inhibitor SR-18,292 (HY-101491) were obtained from MCE (Shanghai, China). Bleomycin hydrochloride was obtained from Hisun Pharm (Taizhou, China). The antibodies used are listed in Table 2.

The MRC-5 cell line was purchased from the American Type Culture Collection (CCL-171) and cultured in MEM with 10% fetal bovine serum, 100 U/mL penicillin, and 100 mg/L streptomycin (P1400; Solarbio, Beijing, China) at 37 °C with 5% CO_2_. The MRC-5 cell line was tested and confirmed to be mycoplasma-free.

### Isolation of primary mouse and human lung fibroblasts

Primary mouse lung fibroblasts were isolated following established procedures(Wan et al. 2023). Lung tissues from 6 to 8 weeks old C57BL/6 N mice were minced and digested with 1 mg/mL collagenase (C0130; Sigma, MO, USA) in Hanks’ balanced salt solution for 30 min, followed by centrifugation at 300 g for 5 min. The obtained cells were washed, resuspended in DMEM (Gibco, Grand Island, NY, USA) medium supplemented with 10% fetal bovine serum, 100 U/mL penicillin, and 100 mg/L streptomycin, and cultured in 10-cm dishes for 7 days. For subsequent experiments, only confluent cells with 3–6 passages were utilized to ensure experimental consistency and reliability.

Primary human lung fibroblasts were isolated following the methods described in the literature(Tsoyi et al. 2021). Lung tissues from the non-diseased marginal areas of lung cancer specimens were minced and digested with 1 mg/mL collagenase in Hanks’ balanced salt solution for 1–2 h, followed by centrifugation at 300 g for 5 min. The obtained cells were washed, resuspended in DMEM medium supplemented with 10% fetal bovine serum, 100 U/mL penicillin, and 100 mg/L streptomycin, and cultured in 10-cm dishes for 10 days. For subsequent experiments, only confluent cells with 3–8 passages were utilized to ensure experimental consistency and reliability.

### Plasmids and transfection

Flag-tagged CAMKK2 was inserted into the pcDNA3.1 vector by a standard subcloning method. Flag-Tagged CAMKK2 R: CCGCTCGAGctactcgggctccatggcctcctcc. F: CCCAAGCTTGCCACCA.

TGGATTACAAGGATGACGACGATAAGtcatcatgtgtctctagccagc. Flag-CAMKK2-pcDNA3.1 or control plasmid (pcDNA3.1) were transfected into MRC-5 cells and primary human lung fibroblasts by Lipofectamine 3000 (L3000015; Invitrogen, Waltham, MA, USA) following the manufacturer’s instructions. Cells were transfected with Flag-CAMKK2-pcDNA3.1 or control plasmid for 24 h, prior to challenge with 5 ng/mL TGF-β1 for 24 h. After 48 h, the cells that were transfected were employed for further experiments.

Silencing CAMKK2 was achieved by targeting the sequences 5^/−^GTGAAGACCATGATACG.

TAAA-3^/^ (shCAMKK2) in the pLKO.1 vector. Concentrate lentivirus particles were used to infect sub-confluent cultures in the presence of 5 µg/mL polybrene overnight following established procedures. Cells were infected with shCAMKK2 or shNC for 24 h, prior to challenge with 5 ng/mL TGF-β1 for 24 h. After 48 h, the cells were employed for further experiments.

### Quantitative real-time PCR (RT-qPCR)

Total RNA was efficiently extracted using TRIzol reagent (15596026CN; Invitrogen, Waltham, MA, USA). Subsequently, Prime Script reverse transcriptase (11141ES60; Yeasen, Shanghai, China) was used for reverse transcribed the RNA into cDNA. qPCR SYBR Green Master Mix (11184ES08; Yeasen, Shanghai, China) was applied following the manufacturer’s instructions precisely. All samples were run in triplicate, with β-actin as the internal reference gene. The relative mRNA expression was calculated via the 2^− ΔΔCt^ method. The primer sequences of target genes are detailed in Table 1.

**Table 1:** The primer sequences used

Gene	Forward primer	Reverse primer
Primers used for qPCR (mouse)		
* Col1a1*	5^/^-GATTCCCTGGACCTAAAGGTGC-3^/^	5^/^ -AGCCTCTCCATCTTTGCCAGCA-3^/^
* Fn1*	5^/^ -ACAACACCGAGGTGACTGAGAC-3^/^	5^/^ -GGACACAACGATGCTTCCTGAG-3^/^
* Acta2*	5^/^ -CTATGCCTCTGGACGCACAACT-3^/^	5^/^ -CAGATCCAGACGCATGATGGCA-3^/^
* Actb*	5^/^ -CATTGCTGACAGGATGCAGAAGG-3^/^	5^/^-CCACAGGATTCCATACCCAAG-3^/^
* Camkk2*	5^/^-TCATGTGTCTCTAGCCAGCC-3^/^	5^/^-TGACCACGATGAAGGATTCCAT-3^/^
Primers used for qPCR (human)		
* FN1*	5^/^ -ACAACACCGAGGTGACTGAGAC-3^/^	5^/^ -GGACACAACGATGCTTCCTGAG-3^/^
* COL1A1*	5^/^ GAGGGCCAAGACGAAGACATC-3^/^	5^/^-CAGATCACGTCATCGCACAAC-3^/^
* ACTA2*	5^/^-CTCTGGACGCACAACTGGCATC-3^/^	5^/^ -TGCTGGAAGGTGGACAGTGAGG-3^/^
* ACTB*	5^/^-CACCATTGGCAATGAGCGGTTC-3^/^	5^/^-AGGTCTTTGCGGATGTCCACGT-3^/^
* CAMKK2*	5^/^-AAGACCAGGCCCGTTTCTACT-3^/^	5^/^-CCAGGAGGTTGGAAGGTTTGA-3^/^

### Western blotting

Cell lysates and lung tissue homogenates were lysed with RIPA lysis buffer containing protease and phosphatase inhibitors (P0013B, P1045; Beyotime, Shanghai, China). After sonication, samples were centrifuged at 10,000×g for 15 min, and the supernatants were collected and boiled at 100 °C for 10 min. Protein concentrations were measured using BCA protein quantification kit (PC0020; Solarbio, Beijing, China). Equal amounts of proteins were separated by 8-12.5% SDS-PAGE, transferred to PVDF membranes (Millipore, Burlington, USA), and then incubated with primary antibodies overnight at 4 °C. Afterward, membranes were incubated with HRP-conjugated secondary antibodies for 1–2 h at room temperature. Protein bands were detected by ECL reagent and quantified using Image J software with β-actin as the internal reference(Li et al. 2024). Antibody details are listed in Table 2.

**Table 2:** The Antibodies and Regents Used

Designation	Source	Category
Antibodies		
COL1A1	Cell Signaling Technology	72026
α-SMA	Abcam	ab5694
FN1	Proteintech	15613-1-AP
COL1A1	Proteintech	14695-1-AP
PGC-1α	Proteintech	66369-1-Ig
FLAG-tag	Proteintech	20543-1-AP
β-Actin	Affinity	AF7018
CAMKK2	Affinity	DF4793
MFN2	Abcam	ab124733
MFN1	Cell Signaling Technology	14739
DRP1	Cell Signaling Technology	8570
Goat anti-Rabbit IgG	Abcam	ab205718
Goat anti-Mouse IgG	Abcam	ab6789
Vimentin	Cell Signaling Technology	5741
p-^T172^AMPK	Cell Signaling Technology	50081
t-AMPK	Cell Signaling Technology	5831
SPC	Affinity	DF6647

### Transwell assay

The treated MRC-5 cells were digested and resuspended in serum-free MEM. A density of 2 × 10⁴ cell suspension was transferred to the upper chamber of transwell inserts, with complete medium in the lower chamber. The transwells in a 24-well plate were incubated at 37 °C in a humidified incubator for 24 h. Migrated cells were fixed with 4% paraformaldehyde for 30 min, stained with crystal violet for 10 min, and then counted under a microscope to evaluate cell migration. Image J was used to quantify the migrating cells.

### Collagen gel contraction assay

Following established protocols, fibroblast contraction assay was conducted(Wan et al. 2023). The treated MRC-5 cells were digested and resuspended in serum-free MEM at a concentration of 3 × 10⁶ cells/mL. The cell suspension was then mixed with 3 mg/mL neutralized rat tail type I collagen (354236; CORNING, USA) at a 1:2 ratio (250µL per well) in a 48-well plate and incubated at 37℃ for 2 h to promote collagen polymerization. After polymerization, 250µL of fresh medium was added to each well, and the cells were further cultured at 37℃ for 48 h. At the end of the culture, the collagen gels were photographed, and the gel contraction rate was calculated as the percentage of the contracted area relative to the initial surface area of the released gel. Image J was used to measure the collagen gel area(Wang et al. 2022).

### Wound-healing assay

Following established protocols, fibroblast wound-healing assay was conducted(Xu et al. 2024). MRC-5 cells were evenly seeded into 6-well plates. After designated treatments, the treated cells reached 90% − 100% confluence, a sterile pipette tip was used to create a straight, uniform scratch across the cell monolayer, simulating a wound injury. PBS was then carefully applied to wash away cellular debris generated during scratching, ensuring a clean experimental field. The scratched areas were imaged at 0 and 24 h. ImageJ was employed to measure the wound width at both time points, enabling a quantitative assessment of cell wound healing efficiency. The wound healing rate was calculated using the formula: [1- (width of the wound at 24 h/width of the wound at time = 0)] ×100%.

### Mito-tracker assay

MRC-5 cells were seeded on glass coverslips in 24-well plates. After designated treatments, cells were gently washed with PBS twice times. Subsequently, the pre-prepared Mito-tracker staining working solution (C1032; Beyotime, Shanghai, China), diluted at a ratio of 1:1000, was added and incubated at 37 °C for 30 min. Then the cell nuclei were stained with DAPI for 5 min. Mitochondrial morphology was visualized using a confocal laser-scanning microscope (Leica, Wetzlar, Germany). To analyze and quantify mitochondrial network morphology/structure the Mina Plugin of Image J software was used in individual cell as described previously (Valente et al. 2017). More details about the image processing can be found here (https://imagej.net/plugins/mina). Thirty cells for each group were used statistical analysis.

### ROS level measurement

Intracellular ROS levels were assessed using a ROS assay kit (S0033S; Beyotime, Shanghai, China). MRC-5 cells were seeded in 96-well plates. After designated treatments, cells were gently washed with PBS twice times. Following a 30 min incubation at 37 °C with 10 µM DCFH-DA, the fluorescence intensity of the cells was measured using a microplate reader at an excitation wave length of 535 nm.

### Mitochondrial membrane potential

Mitochondrial membrane potential (MMP) was assessed using the JC-1 assay kit (C2003; Beyotime, Shanghai, China). Briefly, MRC-5 cells were seeded in 96-well plates and subjected to designated treatments. Following treatment, cells were incubated with JC-1 working solution at 37 °C for 20 min, washed twice with assay buffer, and immediately visualized using fluorescence microscope (Zeiss, Germany). In functional mitochondria with preserved membrane integrity, JC-1 accumulates as red fluorescent aggregates, while loss of MMP results in cytosolic dispersion of green fluorescent monomers. The red-to-green fluorescence intensity ratio was quantitatively analyzed to assess changes in MMP.

### ATP measurement

Cellular ATP levels were measured using the ATP assay kit (S0027, Beyotime, Shanghai, China) according to the manufacturer’s instructions. Briefly, MRC-5 cells were cultured in 6-well plates and treated as indicated. Then cells were lysed and the supernatant was collected following centrifugation. The ATP reaction mixture was then added to the samples and incubated at room temperature for 5 min. Luminescence signals were quantified using a multimode microplate reader (BioTek Synergy LX).

### Seahorse assay

Mitochondrial oxidative phosphorylation was measured by the oxygen consumption rate (OCR) following the methods described in the literature(Yin et al. 2025). Briefly, a density of 2 × 10^4^ cells/well were seeded into XF96 cell culture microplates (103179-100; Agilent, Santa Clara, CA, USA) and transfected. The oxygen consumption rate (OCR) was measured using the Cell Mito Stress Test Kit (103015-100; Agilent, Santa Clara, CA, USA) according to the manufacturer’s instructions.

### Immunofluorescence

MRC-5 cells were seeded on glass coverslips in 24-well plates. After designated treatments, cells were fixed with 4% paraformaldehyde at room temperature for 30 min. Cells or mouse lung tissues sections were permeabilized with 0.3% Triton-100 in PBS for 10 min, and blocked with 1% BSA in PBS for 1 h. Coverslips were incubated with primary antibody at 4 °C overnight, followed by secondary antibodies labeled with Alexa Fluor 488 and/or 594 at room temperature for 1 h. Cell nuclei were stained with DAPI staining reagent (C0065; Solarbio, Beijing, China). Then, coverslips were mounted on slides using an anti-fluorescence quenching mounting medium (S2100; Solarbio, Beijing, China). Images were acquired with a laser confocal microscope (Leica, Wetzlar, Germany).

### Mice

Eight-week-old male C57BL/6 N mice were obtained from Beijing Charles River Laboratory Animal Technology Co., Ltd. (Beijing, China) and housed in a specific pathogen-free environment. All animal experiments were approved by the Institutional Animal Care and Use Committee. Animal care and handling followed Henan Normal University’s IACUC guidelines (IACUC, SMKX-2118BS1018) in accordance with the Association of Animal Behavior and national regulations. The mice were acclimated for a week prior to intratracheal administration of 1 × 10¹¹ vg per mouse AAV6-Camkk2 or AAV6-NC (OBiO Technology Corp., Ltd.). The selected dose (1.5 × 10^13^ vg/ml) was based on the titration experiments showing optimal transduction with minimal toxicity by the biotechnology company (OBiO Technology Corp., Ltd.). For bleomycin induced pulmonary fibrosis(Pan et al. 2024), mice were anesthetized with isoflurane, and 1.7 U/kg bleomycin in 50 µL saline was administered intratracheally on Day 0. Control mice received 50 µL saline in the same manner. Mice were sacrificed on Day 14, and lung tissues and BAL were collected for histological, collagen, and biochemical analyses.

### Hydroxyproline

Collagen quantification was assessed using a hydroxyproline assay kit (AO303-1; Nanjing Jiancheng, Nanjing, China) following the manufacturer’s instructions. First, 100 mg of minced lung tissue was accurately weighed and hydrolyzed with 1 ml of 6 mol/L HCl in a boiling-water bath for 5 h. Subsequently, the pH of the hydrolysate was adjusted to a range of 6.0-6.8. Then, approximately 30 mg of activated carbon was added to 3 ml of the diluted test solution, which was thoroughly mixed and centrifuged at 3500 rpm for 10 min. One milliliter of the supernatant was carefully collected. Next, 0.5 ml each of Reagent 1, Reagent 2, and Reagent 3 was added to the supernatant, and the mixture was well-mixed. The mixture was then incubated in a 60℃-water bath for 15 min, cooled down, centrifuged again at 3500 rpm for 10 min, and the absorbance of the supernatant was measured at 550 nm.

### Hematoxylin and Eosin (H&E) and Masson staining

Hematoxylin and eosin (H&E) staining and Masson’s trichrome staining were carried out following the methods described in the literature(Wan et al. 2023). Mouse lung tissues were fixed in 4% paraformaldehyde for 24 h, dehydrated, and embedded in paraffin. Subsequently, 4-µm-thick sections were prepared and deparaffinized in distilled water via standard procedures(Li et al. 2025). To evaluate morphological changes, both H&E and Masson’s trichrome staining were performed using commercial kits (G1120, G1340; Solarbio, Beijing, China) strictly following the manufacturer’s protocols. Images were acquired using an Olympus BX43 upright microscope (Tokyo, Japan).

### Immunohistochemistry (IHC)

IPF and BLM-treated mouse lung sections were prepared to stain for CAMKK2. Tissue sections were first deparaffinized with xylene, hydrated through graded ethanol solutions, and rinsed with deionized water. After antigen retrieval, endogenous peroxidase activity was blocked by incubating the sections in 3% hydrogen peroxide for 30 min at room temperature. Then, the sections were blocked with 10% normal goat serum for 1 h. The primary antibody was incubated overnight at 4 °C, followed by 60 min incubation with an HRP-labeled secondary antibody at room temperature. Finally, the sections were developed with DAB and counterstained with hematoxylin to complete the staining process. Images were acquired using an Olympus BX43 upright microscope.

### Micro-CT imaging

Micro-CT images (SkyScan 1276, Belgium) of the whole lung were performed on the last survival day. Briefly, mice were Lightly anesthetized with isoflurane and placed in a supine position. Micro - CT images were acquired using the Bruker SkyScan 1276 system. Scanning parameters were precisely set: 60 kV X-ray tube voltage, 200 µA anode current, and a 0.5-mm copper filter. The approximately 15 min acquisition process yielded high quality 3D images for subsequent analysis. The reconstructed images were subjected to overlay processing using the NRecon software (Bruker-microCT, Belgium).

### Statistical analysis

The Shapiro-Wilk test was employed to assess normality. The Mann-Whitney U test was employed for non-normally distributed data, whereas normally distributed data were evaluated with an unpaired Student’s t-test. One-way ANOVA followed by Turkey’s multiple comparison test was employed to compare three or more groups. In vivo efficacy experiments were conducted with at least six mice per group and were independently performed twice. All in vitro experiments were independently replicated at least three times. All data are expressed as mean ± SD, and statistical significance set at *P* < 0.05.

## Supplementary Information

Supplementary Material 1.

Supplementary Material 2.

Supplementary Material 3.

Supplementary Material 4.

Supplementary Material 5.

## Data Availability

All data analyzed are included in the article. Transcriptomic microarray data and differentially expressed gene profiles associated with UIP in human subjects were obtained from the GSE47460 dataset available in the Gene Expression Omnibus (GEO) database ([https://www.ncbi.nlm.nih.gov/geo/](https://www.ncbi.nlm.nih.gov/geo)).

## Abbreviations

IPF: Idiopathic pulmonary fibrosis
ECM: Extracellular matrix
CAMKK2: Calcium/calmodulin dependent protein kinase kinase 2
AMPK: AMP-activated protein kinase
PGC-1α: Peroxisome proliferator-activated receptor γ coactivator 1α
MFN1: Mitofusin protein 1
MFN2: Mitofusin protein 2
DRP1: Dynamin-related protein 1
ROS: Reactive oxygen species
MMP: Mitochondrial membrane potential
OCR: Oxygen consumption rate
OXPHOS: Oxidative phosphorylation
COL1A1: Type I collagen alpha 1 chain
α-SMA: Alpha-smooth muscle actin
IHC: Immunohistochemistry
ND1: NADH dehydrogenase subunit 1
CYTB: Cytochrome b
MT-CO2: Mitochondrially-encoded cytochrome c oxidase II
ATP8: ATP synthase F0 subunit 8
AEC II: Alveolar type II epithelial cell
UIP: Usual interstitial pneumonia
