# Supplementary material for: CAMKK2 restored mitochondrial dynamics homeostasis to alleviate pulmonary fibrosis via AMPK/PGC-1α signaling pathway in lung fibroblasts
Source: Mol Med. 2025 Oct 6;31:308. doi: 10.1186/s10020-025-01373-5 (PMC12502149; doi:10.1186/s10020-025-01373-5)
Supplement: Supplementary file 1 — Supplementary Material 1. [file 10020_2025_1373_MOESM1_ESM.pdf]

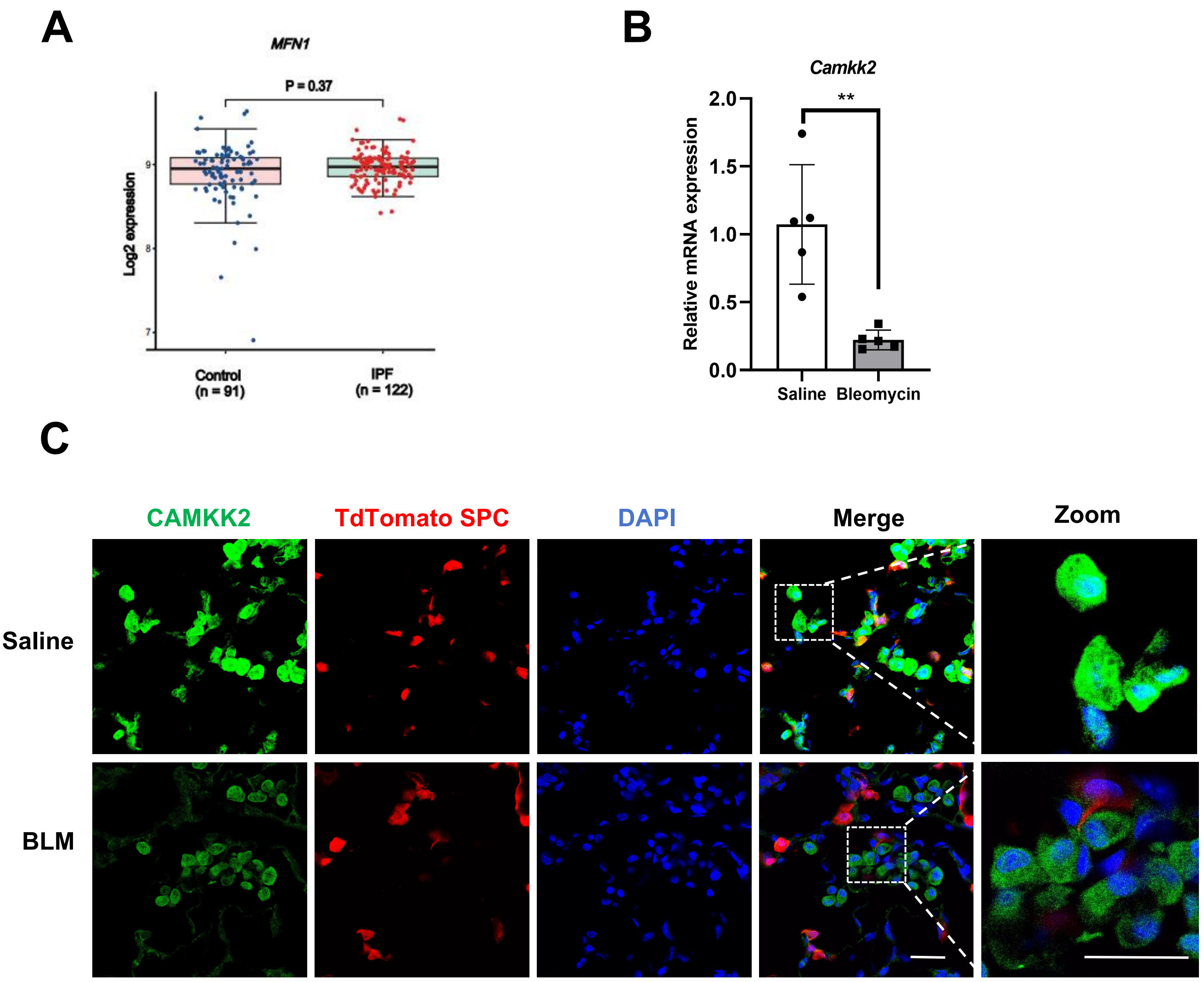

Fig. S1 A RNA expression of the *MFN1* in the GSE47460 microarray datasets. **B** RT-qPCR analysis of *Camkk2 expression* in mouse lung homogenates (n=5). **C** Representative images of co-staining of CAMKK2 with SPC (an alveolar type II epithelial cell marker) in lung tissues from bleomycin induced mice and saline control (n=5). Scale bars = 50µm. The values are shown as mean ± SD. *$P < 0.05$; **$P < 0.01$; ***$P < 0.001$.
