# Supplementary material for: CAMKK2 restored mitochondrial dynamics homeostasis to alleviate pulmonary fibrosis via AMPK/PGC-1α signaling pathway in lung fibroblasts
Source: Mol Med. 2025 Oct 6;31:308. doi: 10.1186/s10020-025-01373-5 (PMC12502149; doi:10.1186/s10020-025-01373-5)
Supplement: Supplementary file 2 — Supplementary Material 2. [file 10020_2025_1373_MOESM2_ESM.pdf]

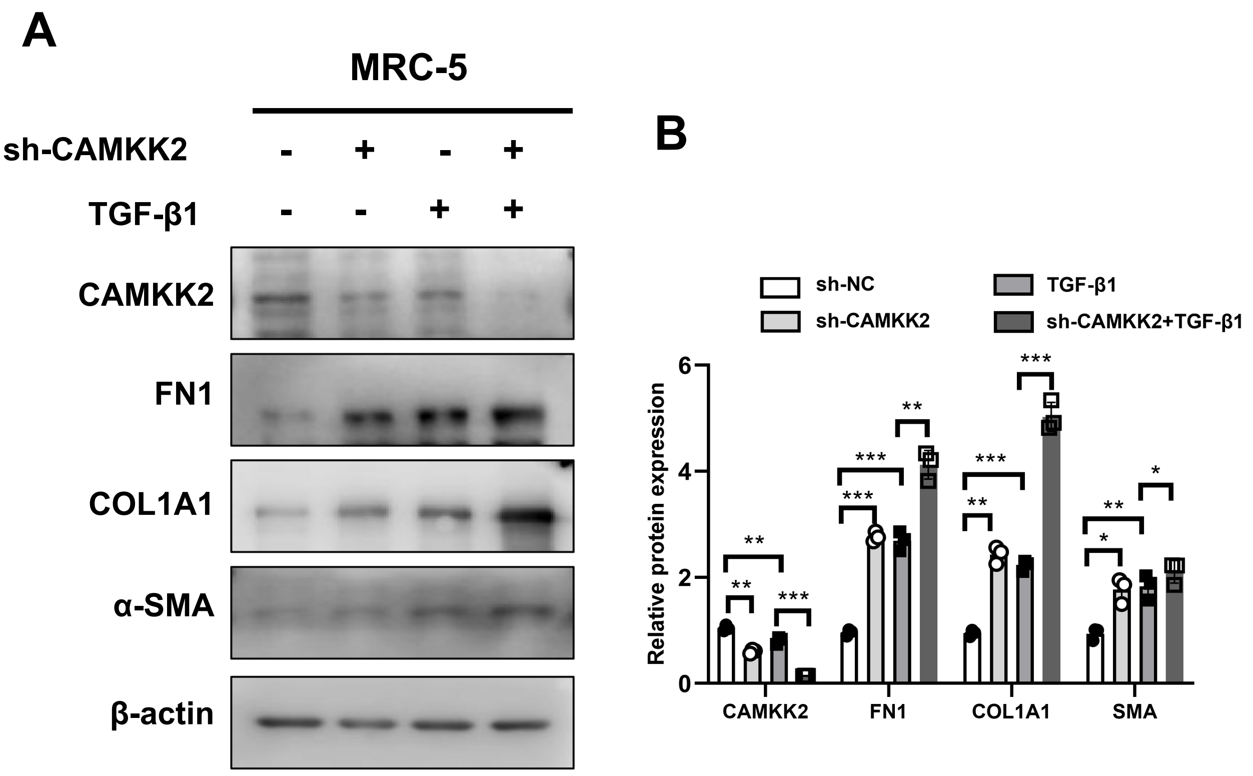

Revised Fig. S2 A-B Western blotting analysis and quantification of CAMKK2, FN1, COL1A1 and α-SMA levels in MRC-5 cells pretreated with sh-CAMKK2 or sh-NC for 24 h, then stimulated with and without TGF-β1 (5 ng/mL) for 24 h (n= 3). The values are shown as mean ± SD. *$P < 0.05$; **$P < 0.01$; ***$P < 0.001$.
