# Supplementary material for: CAMKK2 restored mitochondrial dynamics homeostasis to alleviate pulmonary fibrosis via AMPK/PGC-1α signaling pathway in lung fibroblasts
Source: Mol Med. 2025 Oct 6;31:308. doi: 10.1186/s10020-025-01373-5 (PMC12502149; doi:10.1186/s10020-025-01373-5)
Supplement: Supplementary file 3 — Supplementary Material 3. [file 10020_2025_1373_MOESM3_ESM.pdf]

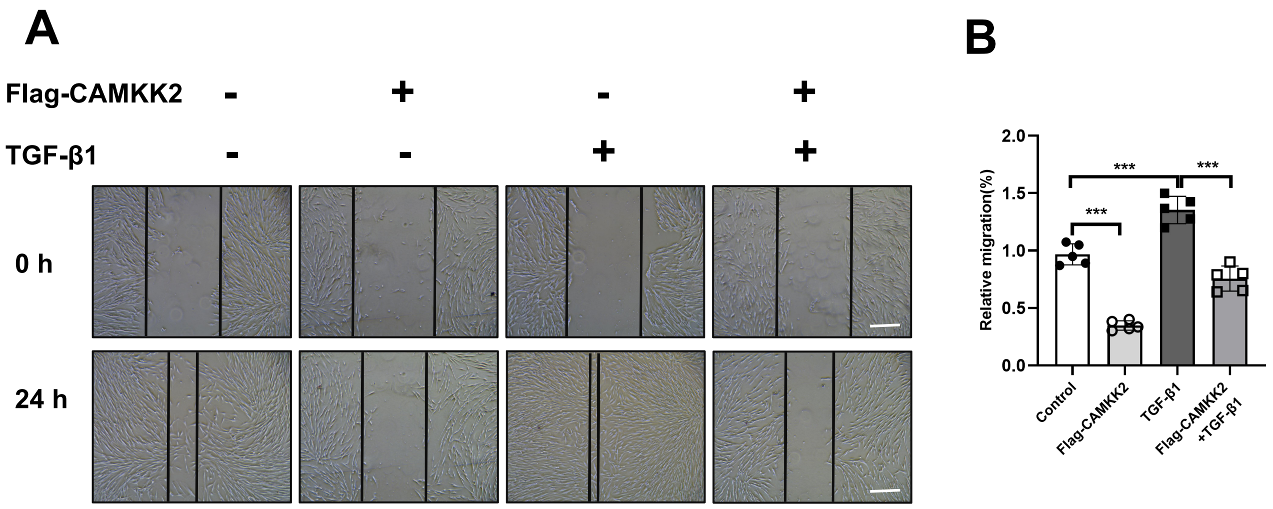


**Fig. S3 A-B** Representative images and quantification of migration activity by wound-healing assay in MRC-5 cells transfected with pcDNA3.1 plasmid or pcDNA3.1-Flag-CAMKK2 plasmid for 24 h, then stimulated with and without TGF-β1 (5 ng/mL) for 24 h (n=5). Scale bars = 100μm. The values are shown as mean ± SD. **P* < 0.05; ***P* <0.01; ****P* < 0.001; NS = not significant.
