# Supplementary material for: CAMKK2 restored mitochondrial dynamics homeostasis to alleviate pulmonary fibrosis via AMPK/PGC-1α signaling pathway in lung fibroblasts
Source: Mol Med. 2025 Oct 6;31:308. doi: 10.1186/s10020-025-01373-5 (PMC12502149; doi:10.1186/s10020-025-01373-5)
Supplement: Supplementary file 4 — Supplementary Material 4. [file 10020_2025_1373_MOESM4_ESM.pdf]

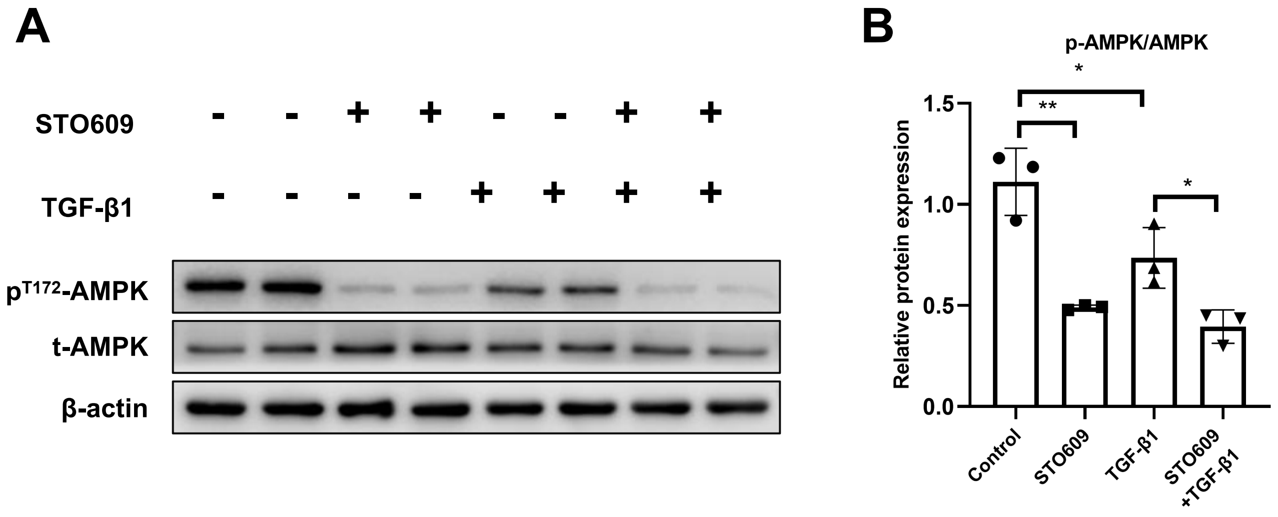


**Fig. S4 A-B** Western blotting analysis and quantification of p-AMPK, and t-AMPK levels in MRC-5 cells pretreated with STO-609 (a selective CAMKK2 inhibitor, 10μM) or empty control for 24 h, then stimulated with and without TGF-β1 (5 ng/mL) for 24 h (n= 3). The values are shown as mean ± SD. **P* < 0.05; ***P* <0.01; ****P* < 0.001; NS = not significant.
