# Supplementary material for: CAMKK2 restored mitochondrial dynamics homeostasis to alleviate pulmonary fibrosis via AMPK/PGC-1α signaling pathway in lung fibroblasts
Source: Mol Med. 2025 Oct 6;31:308. doi: 10.1186/s10020-025-01373-5 (PMC12502149; doi:10.1186/s10020-025-01373-5)
Supplement: Supplementary file 5 — Supplementary Material 5. [file 10020_2025_1373_MOESM5_ESM.pdf]

Full uncropped original gel

Figure 1H

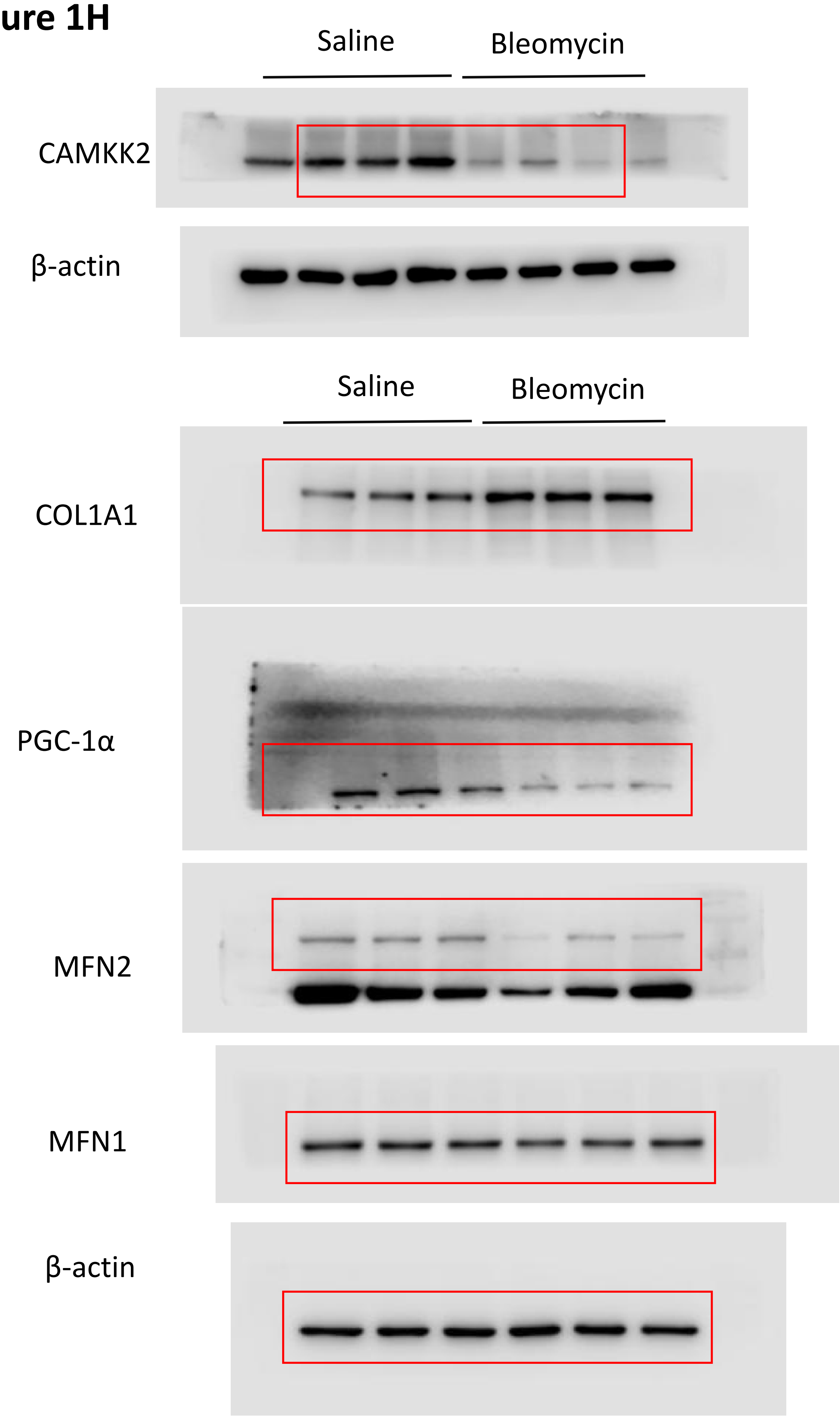

Figure 1J

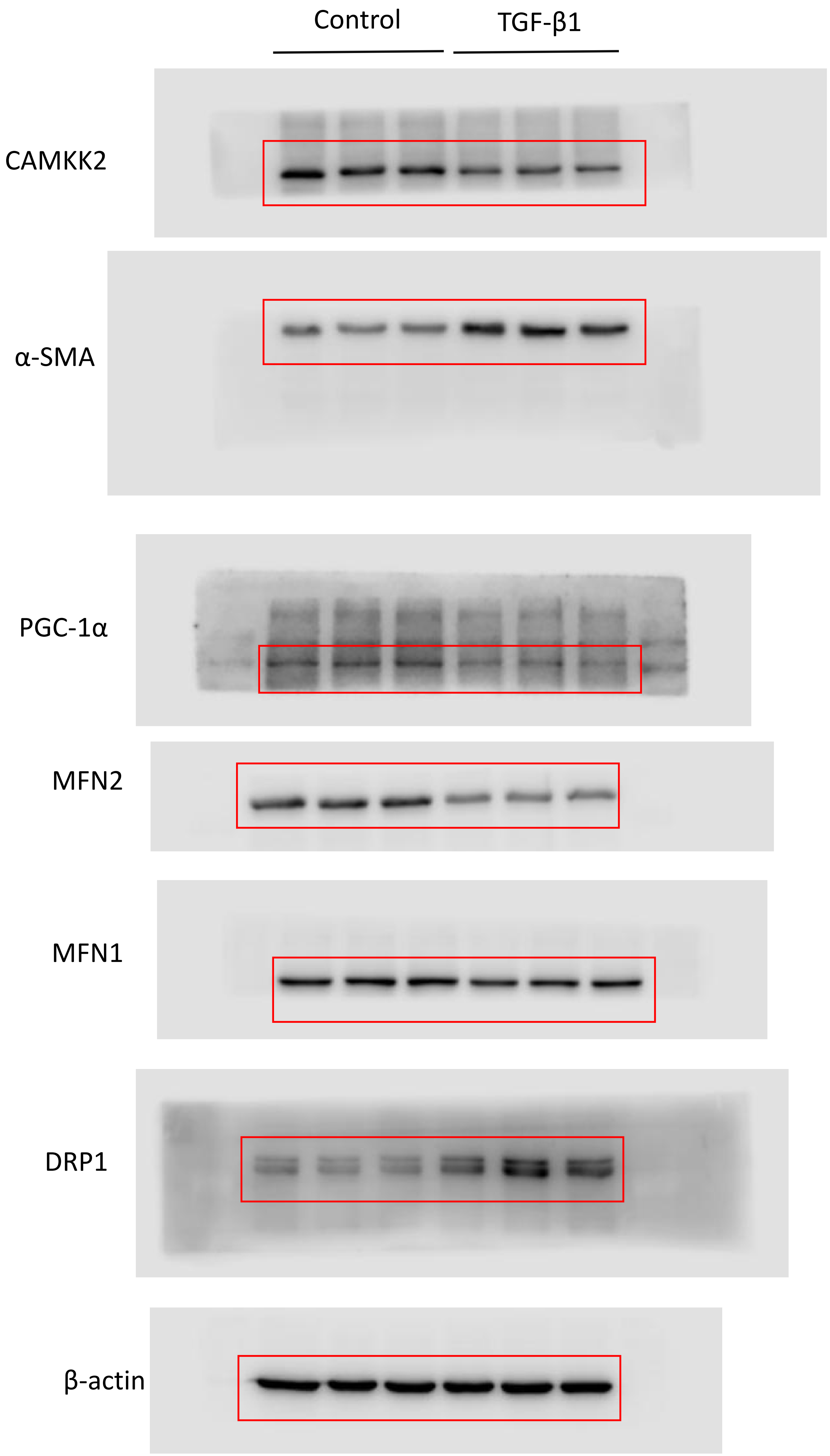

Figure 2B

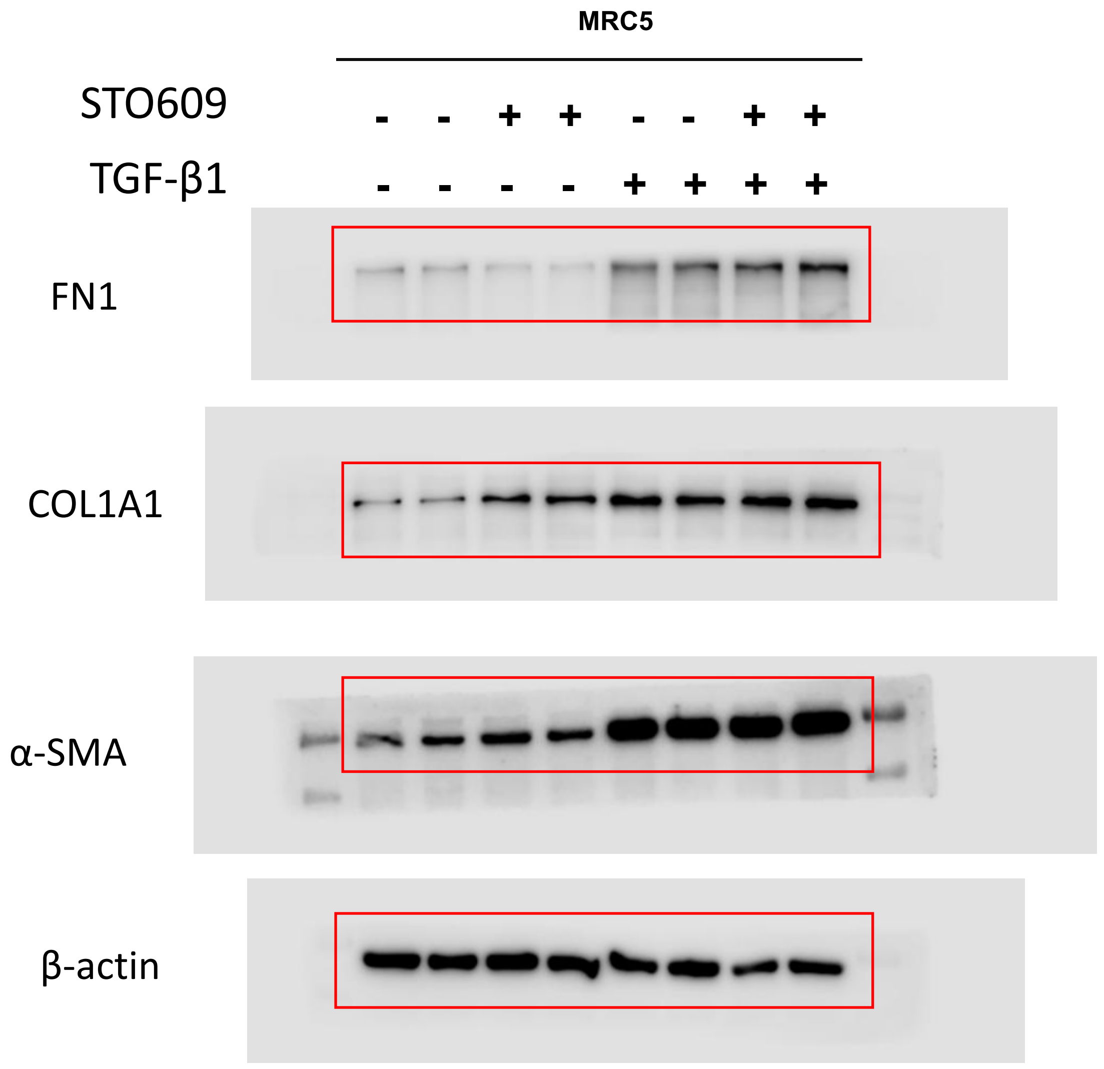

Figure 2H

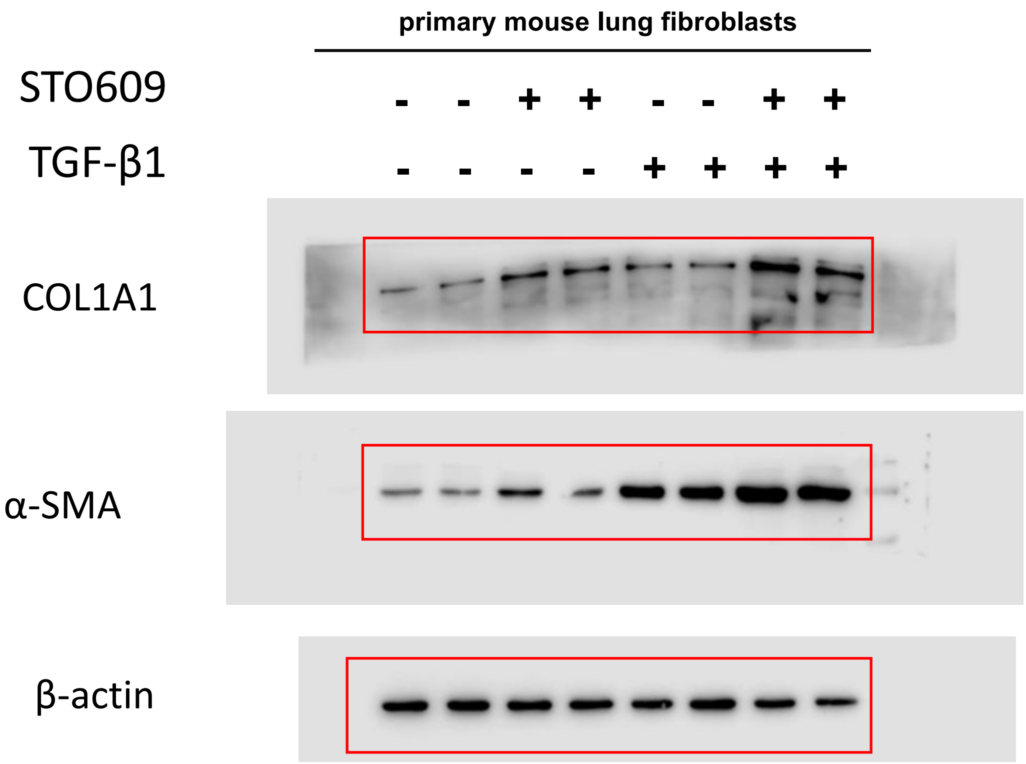
primary mouse lung fibroblasts
STO609 - - + + - - + +
TGF-β1 - - - - + + + +
COL1A1
α-SMA
β-actin

Figure 3B

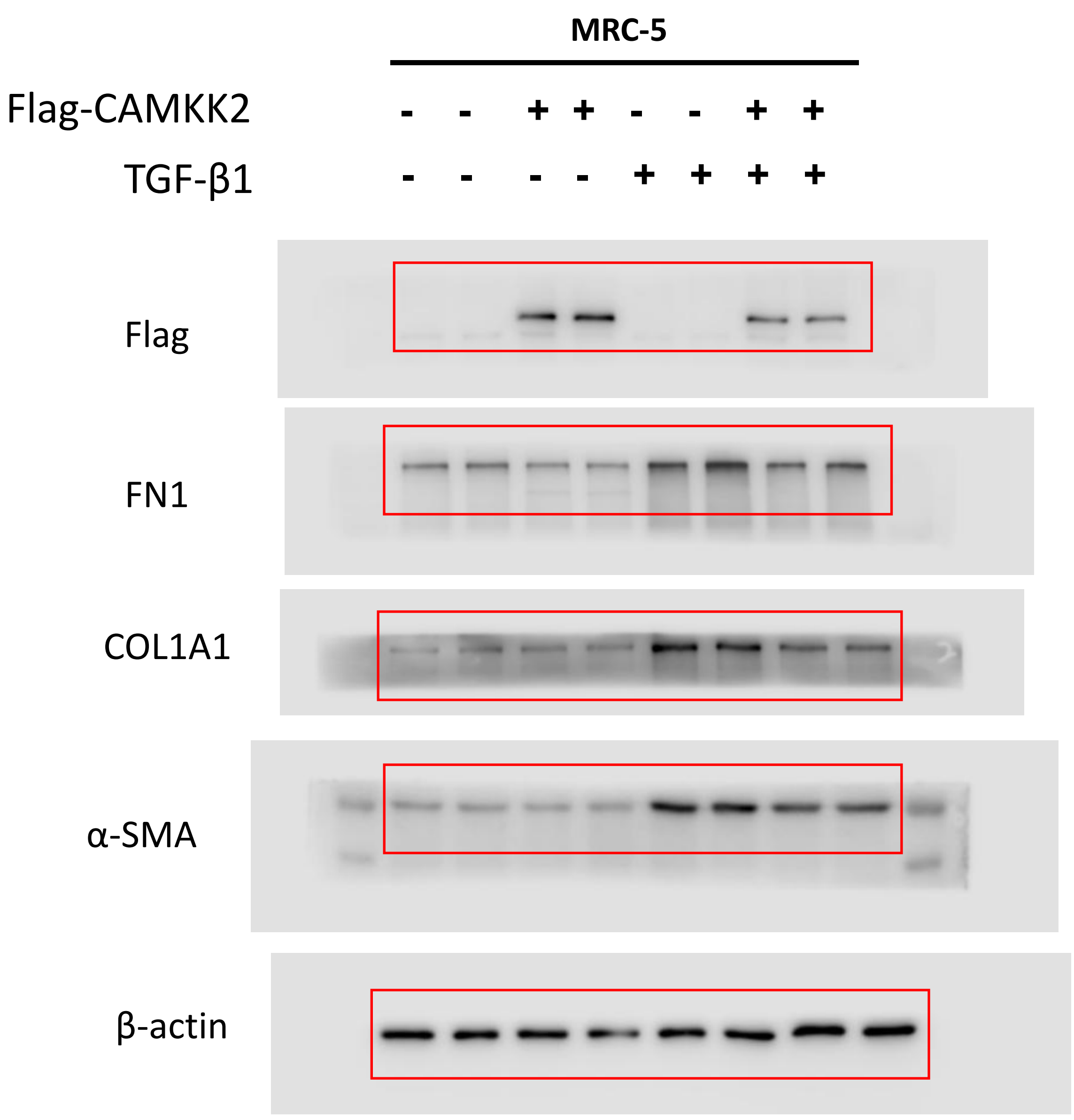

Figure 3H

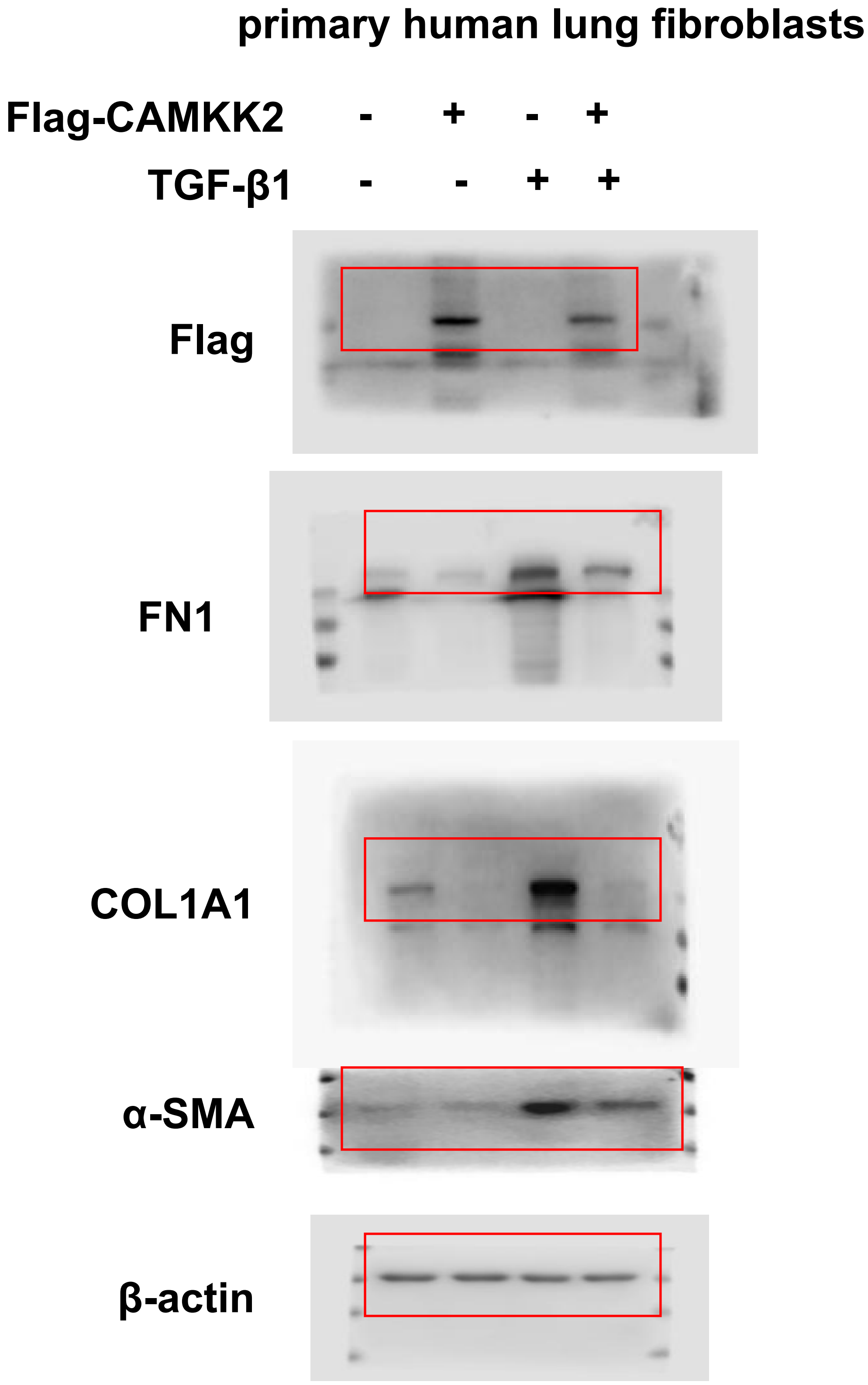
primary human lung fibroblasts
Flag-CAMKK2 - + - +
TGF-β1 - - + +
Flag
FN1
COL1A1
α-SMA
β-actin

Figure 4H

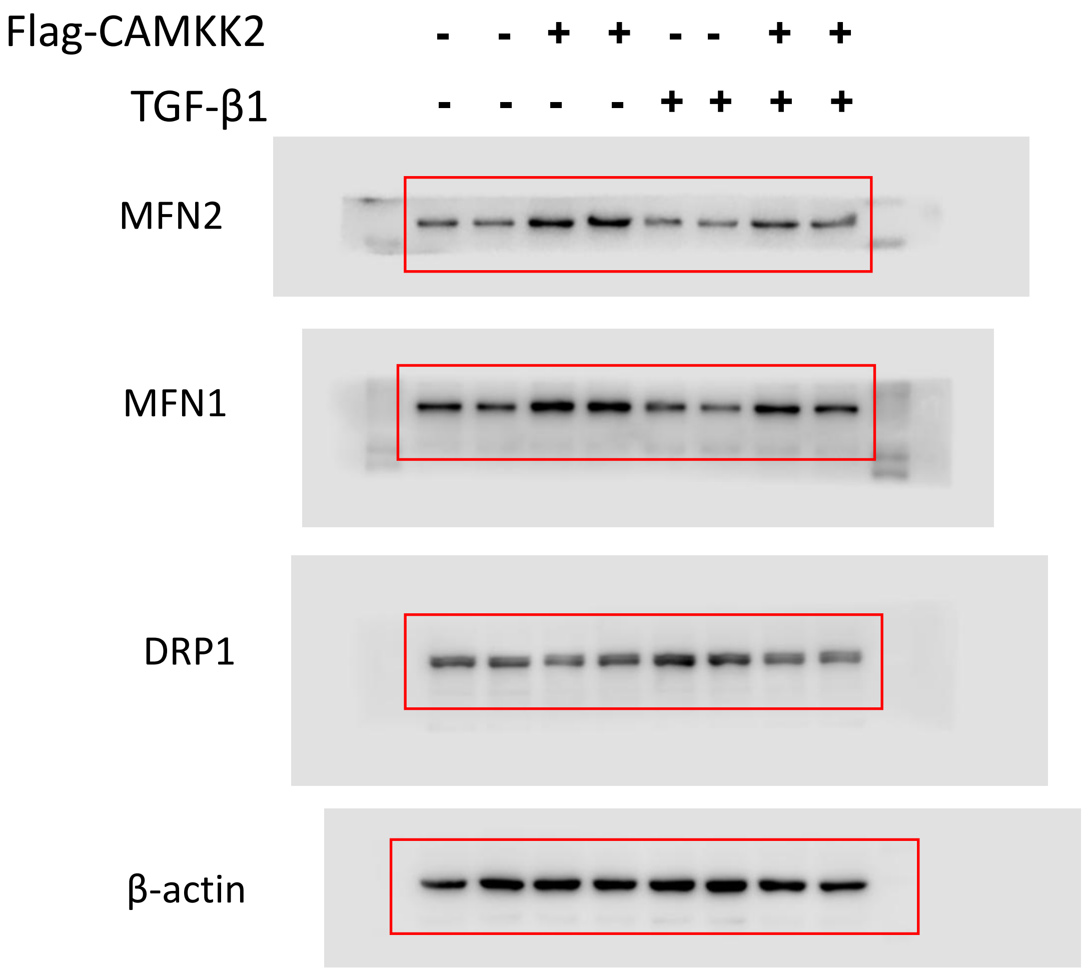

Figure 5A

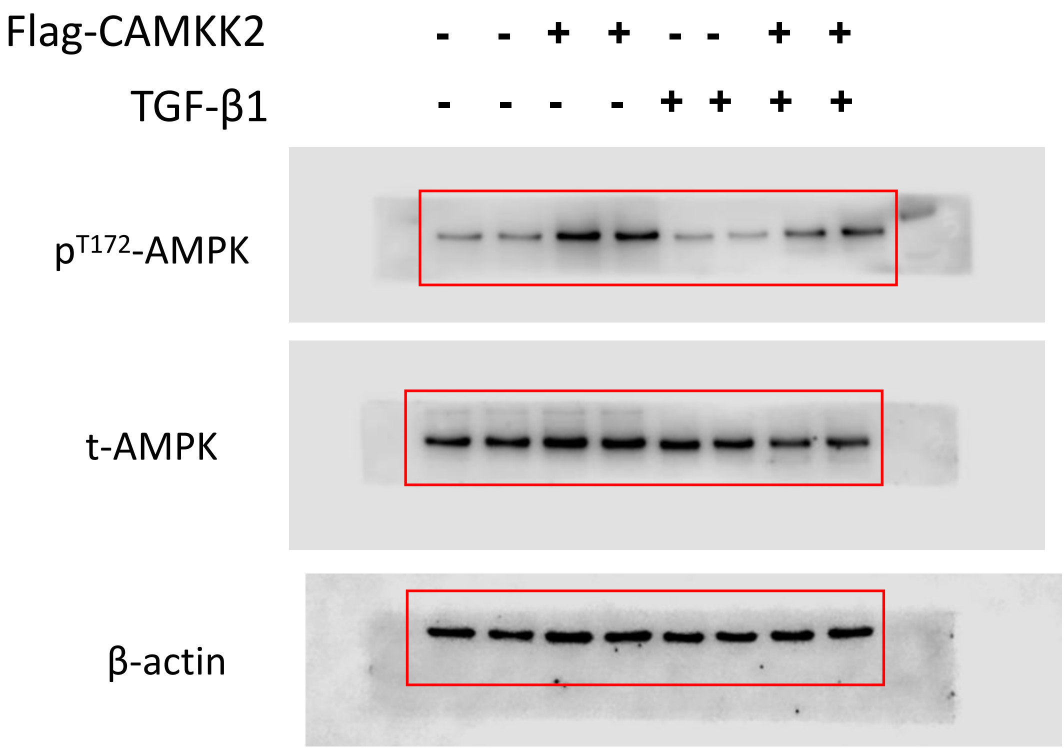

Figure 5C

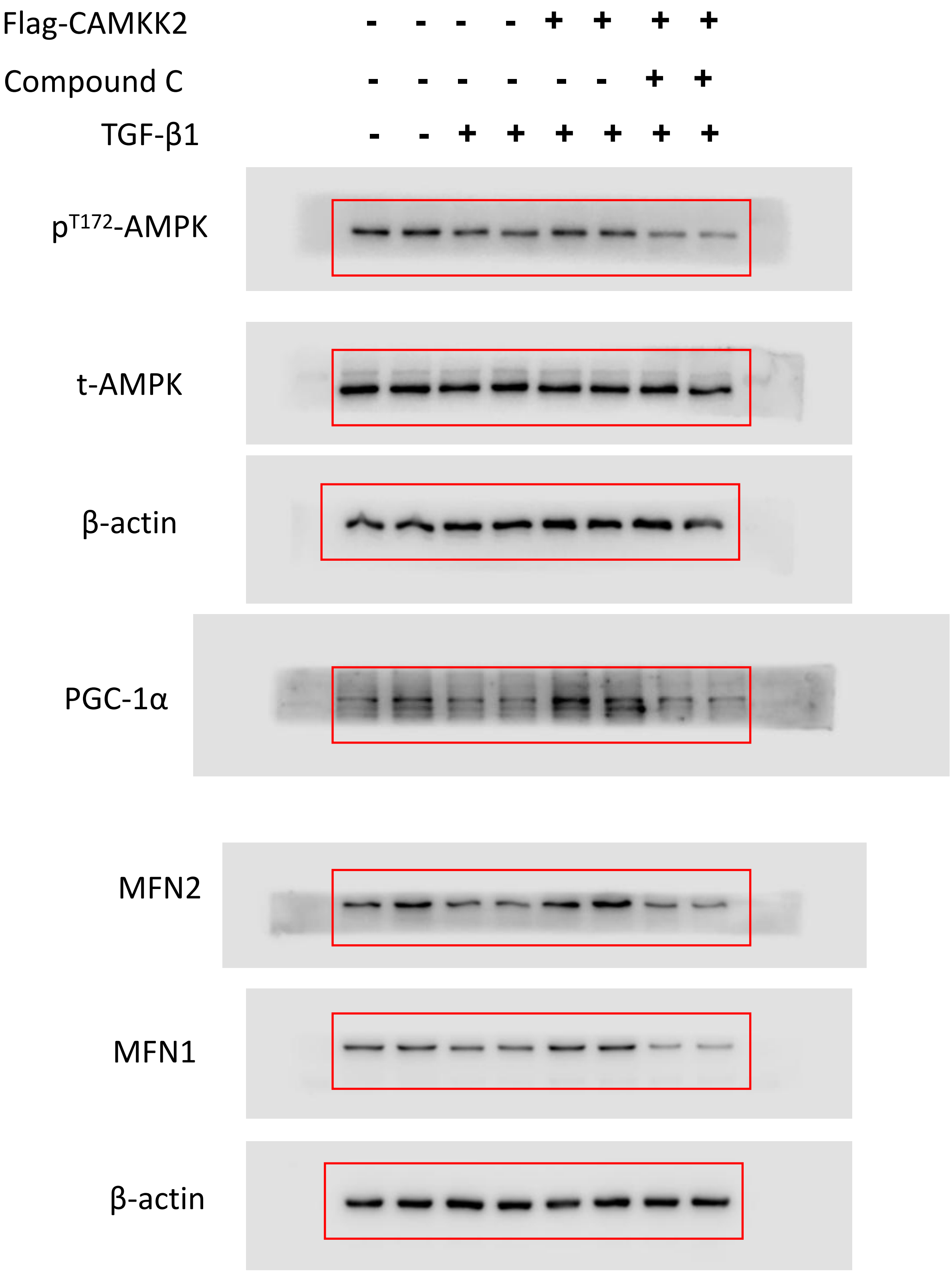
Flag-CAMKK2 - - - - + + + +
Compound C - - - - - - + +
TGF-β1 - - + + + + + +
pT172-AMPK
t-AMPK
β-actin
PGC-1α
MFN2
MFN1
β-actin

Figure 5E

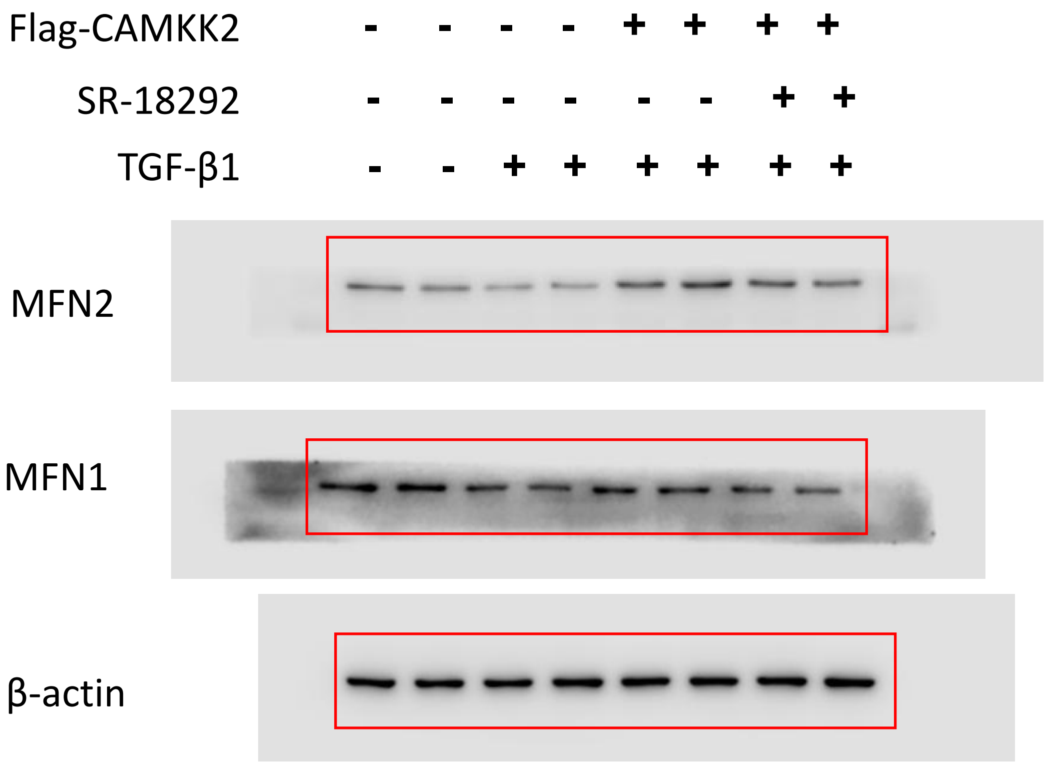

Figure 5K

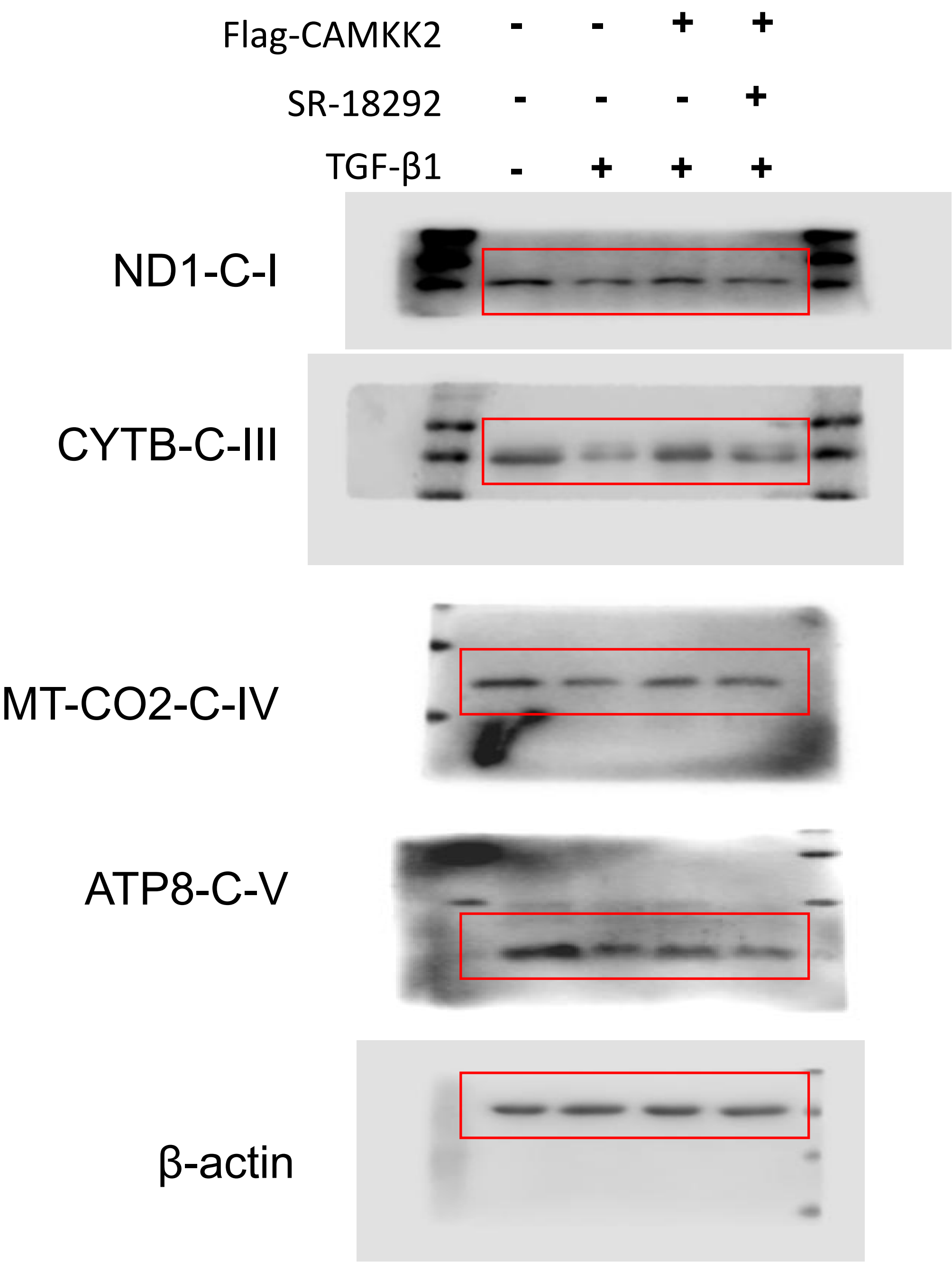

Flag-CAMKK2 - - + +
SR-18292 - - - +
TGF-β1 - + + +
ND1-C-I
CYTB-C-III
MT-CO2-C-IV
ATP8-C-V
β-actin

Figure 5M

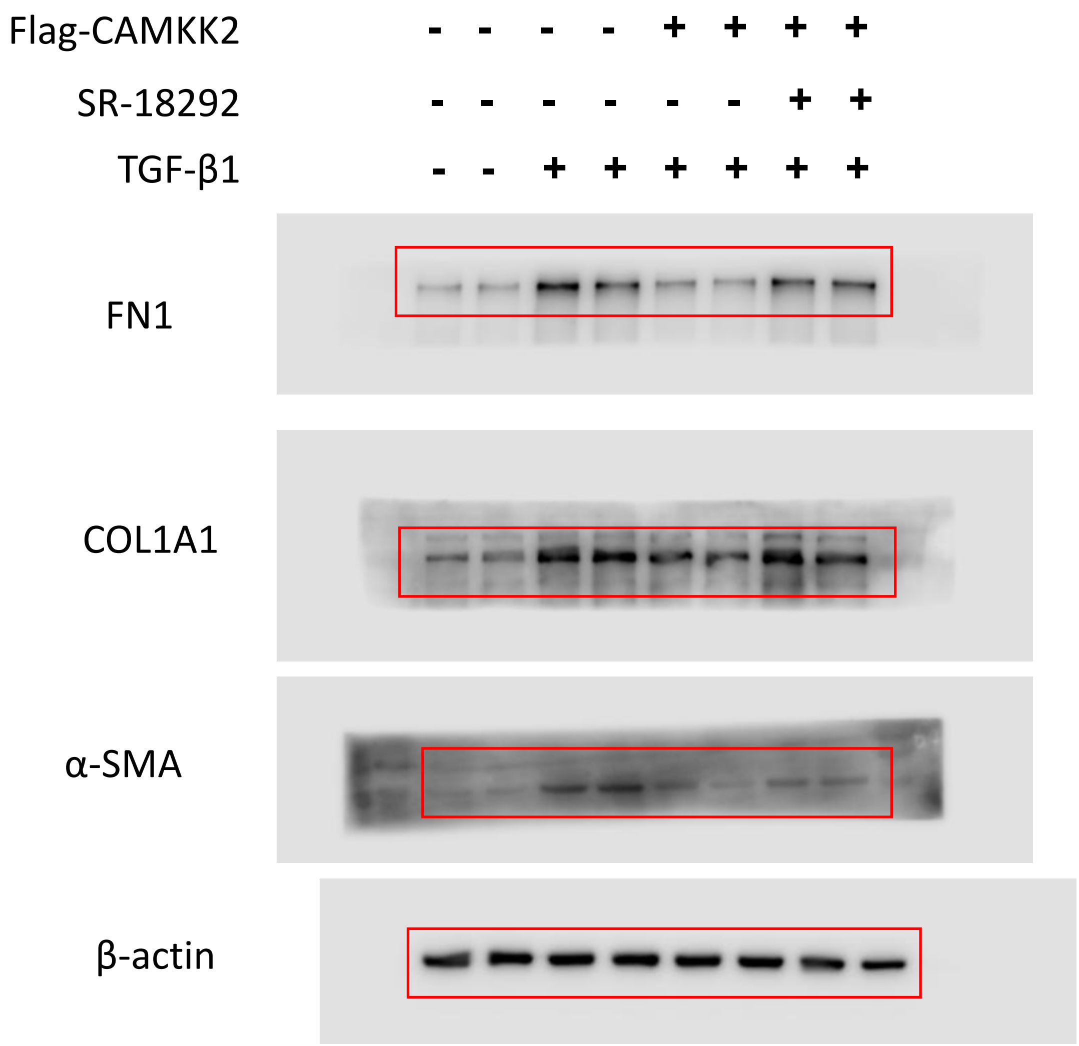

Figure 6J

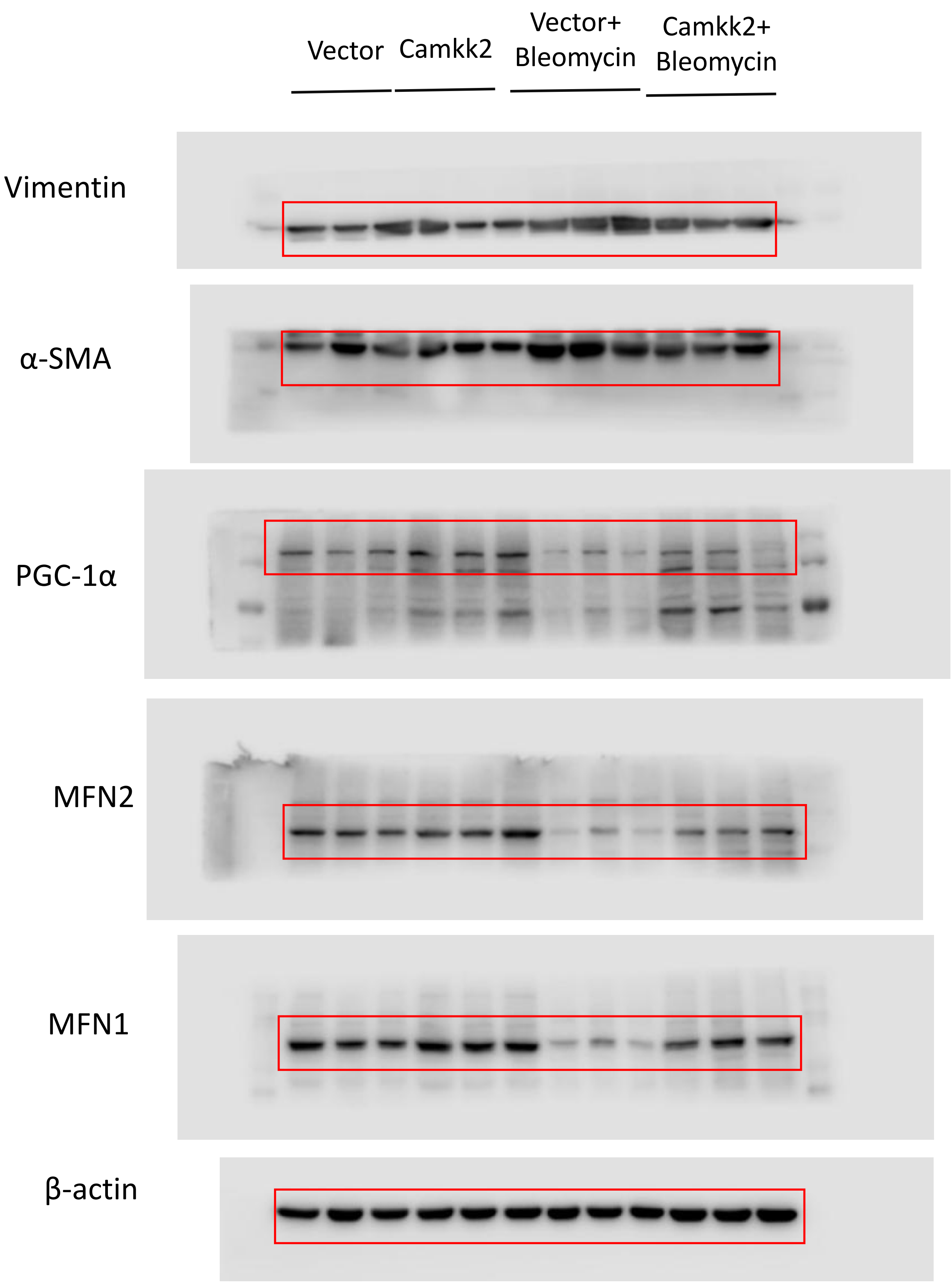

Vector
Camkk2
Vector+ Bleomycin
Camkk2+ Bleomycin
Vimentin
α-SMA
PGC-1α
MFN2
MFN1
β-actin
